# High-fidelity self-assembly pathways for hydrogen-bonding molecular semiconductors

**DOI:** 10.1038/srep43098

**Published:** 2017-02-22

**Authors:** Xu Lin, Mika Suzuki, Marina Gushiken, Mitsuaki Yamauchi, Takashi Karatsu, Takahiro Kizaki, Yuki Tani, Ken-ichi Nakayama, Mitsuharu Suzuki, Hiroko Yamada, Takashi Kajitani, Takanori Fukushima, Yoshihiro Kikkawa, Shiki Yagai

**Affiliations:** 1 Graduate School of Engineering, Chiba University, 1-33 Yayoi-cho, Inage-ku, Chiba 263-8522, Japan; 2 Graduate School of Science and Engineering, Yamagata University, 4-3-16 Jonan, Yonezawa, Yamagata 992-8510, Japan; 3 Graduate School of Engineering, Osaka University, 2-1 Yamadaoka, Suita, Osaka 565-0871, Japan; 4 Graduate School of Material Science, Nara Institute of Science and Technology (NAIST), 8916-5 Takayama-cho, Ikoma, Nara 630-0192, Japan; 5 Laboratory for Chemistry and Life Science, Institute of Innovative Research, Tokyo Institute of Technology, 4259 Nagatsuta, Midori-ku, Yokohama 226-8503, Japan; 6 RIKEN SPring-8 Center, 1-1-1 Kouto, Sayo, Hyogo 679-5148, Japan; 7 National Institute of Advanced Industrial Science and Technology (AIST), 1-1-1 Higashi, Tsukuba, Ibaraki 305-8562, Japan

## Abstract

The design of molecular systems with high-fidelity self-assembly pathways that include several levels of hierarchy is of primary importance for the understanding of structure-function relationships, as well as for controlling the functionality of organic materials. Reported herein is a high-fidelity self-assembly system that comprises two hydrogen-bonding molecular semiconductors with regioisomerically attached short alkyl chains. Despite the availability of both discrete cyclic and polymeric linear hydrogen-bonding motifs, the two regioisomers select one of the two motifs in homogeneous solution as well as at the 2D-confined liquid-solid interface. This selectivity arises from the high directionality of the involved hydrogen-bonding interactions, which renders rerouting to other self-assembly pathways difficult. In thin films and in the bulk, the resulting hydrogen-bonded assemblies further organize into the expected columnar and lamellar higher-order architectures via solution processing. The contrasting organized structures of these regioisomers are reflected in their notably different miscibility with soluble fullerene derivatives in the solid state. Thus, electron donor-acceptor blend films deliver a distinctly different photovoltaic performance, despite their virtually identical intrinsic optoelectronic properties. Currently, we attribute this high-fidelity control via self-assembly pathways to the molecular design of these supramolecular semiconductors, which lacks structure-determining long aliphatic chains.

Precise control over the self-assembly pathway of supramolecular building blocks^1^,^2^ with a π-conjugated core (henceforth: supramolecular π-building blocks) is crucial for the generation of specifically organized structures with desirable optoelectronic properties^3^. Due to the structural complexity in supramolecular π-building blocks that may favor specific conformations for a set of given conditions^4^,^5^,^6^,^7^, it still remains challenging to control their self-assembly pathways, which may include several levels of hierarchy from dilute solution to the bulk^8^,^9^. One of the structural elements that can lead to more complex pathways are long aliphatic chains, which endow the supramolecular π-building blocks with solubility and processability, as well as the resulting assemblies with softness^10^,^11^,^12^. These flexible units not only increase the kinetic stability of the assembled structures under specific conditions, but also influence thermodynamically stable structures during their transfer from solution to the bulk^4^,^13^,^14^,^15^. Moreover, a thermal treatment in the bulk may further affect the self-assembled structures by transferring the aliphatic chains into the molten state^16^,^17^.

In this context, the design of solution-processable supramolecular π-building blocks that do not contain long aliphatic chains should be promising not only from a practical perspective^18^,^19^,^20^, but also with regard to the high probability of generating the desired hierarchically organized architectures via high-fidelity self-assembly pathways. Such a design should be particularly effective for systems that are based on directional hydrogen-bonding interactions, where usually a drastic structural alteration is induced by the rearrangement of the hydrogen-bonding motif upon increasing the level of self-assembly hierarchy or by applying external stimuli^21^,^22^,^23^,^24^. Herein, we report high-fidelity self-assembly pathways of two hydrogen-bonding supramolecular oligothiophene π-building blocks^25^,^26^,^27^,^28^ with regioisomerically attached short alkyl side chains. As the short alkyl chains in these molecules are grafted onto the π-conjugated backbone, they are embedded within the supramolecular structure, which endows the resulting supramolecular assemblies with good solubility. Such regioisomerically attached alkyl chains also give rise to different molecular conformations due to steric demand, which results in a thermodynamic preference for either cyclic or linear hydrogen-bonding motifs. Remarkably, the supramolecular structures formed in solution are preserved upon removal of the solvent, thus yielding predictably organized solid supramolecular architectures. These results demonstrate that control over high-fidelity pathways may substantially impact supramolecular architectures in the solid state, especially upon co-organization with structurally different molecular components for applications in photovoltaic devices.

## Results and Discussion

### Regioisomers

**Figure 1:**
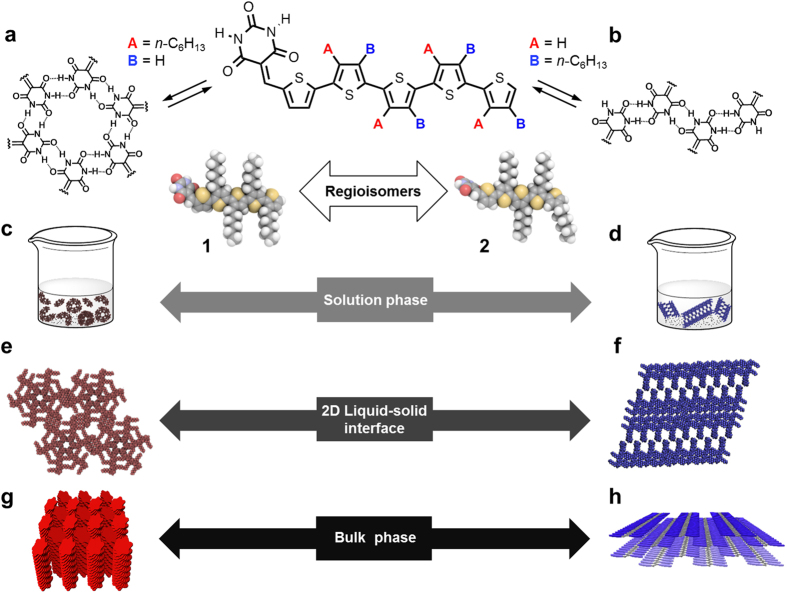
Hierarchical self-assembly of 1 and 2 in different phases. (**a**,**b**) Chemical structures and molecular models of hydrogen-bonding regioisomeric semiconductors **1** and **2** and their different hydrogen bonding motifs. (**c**–**h**) Schematic representation of the hierarchical organization of **1** and **2** in different phases.

Regioisomeric quinquethiophenes **1**^20^ and **2** with barbituric acid group are purple dyes that exhibit an absorption maximum at ~550 nm in CHCl_3_ (Fig. 1a, see Supplementary Fig. S11). Despite similarity in their molecular structures, **1** is significantly more soluble in CHCl_3_ (400 mM, *i.e.* ~355 mg/mL) than **2** (100 mM, *i.e.* ~89 mg/mL). Furthermore, the melting point (m.p.) of **1** (145 °C, 2nd heating scan in a differential scanning calorimetry (DSC) measurement) is lower than that of **2** (165 °C, 2nd heating scan in a DSC measurement). We thus expected **1** and **2** to self-organize in different supramolecular structures via self-complementary hydrogen bonds.

### Self-assembly in solution

Because of the absence of reasonable multicomponent self-assembly systems that provide both cyclic and polymeric hydrogen-bonded aggregates soluble without forming higher-order assemblies, the structural preference for these competing hydrogen-bonded motifs have been mainly studied based on solid-state packing structures provided by X-ray crystallography^29^ or theoretical approaches^30^. Following vapor pressure osmometry (VPO)^31^,^32^,^33^ and nuclear magnetic resonance (NMR) studies of **1** and **2** in chloroform however reveal such a structural preference could occur in equilibrated solution state.

VPO measurements of chloroform solutions of **1** and **2** at substrate concentrations (*c*_subs_) from 10 to 100 mM displayed markedly different concentration-dependence on aggregation (see Supplementary Fig. S12). For **1**, the average aggregation number *N* = *c*_subs_/*c*_collig_, wherein *c*_collig_ is the colligative concentration measured by VPO, increased from 1.3 to 2.1 upon increasing concentration from 10 mM to 100 mM. Though the results are indicative of the formation of dimers, the selective formation of dimers was clearly discarded on account of the following concentration-dependent NMR study that illustrate the formation of larger oligomers including cyclic hexamers (rosettes). Furthermore, the presence of residual monomers (ca. 20%) in the monomer-aggregate equilibrium could seriously influence *c*_collig_, leading to this underestimation of *N*^32^. On the contrary, **2** exhibited a linear increase of *N* with increasing concentration, and *N* reached 9.9 for *c* = 100 mM. This tractable concentration-dependence of *N* is in good agreement with the formation of open-ended oligomers. For both compounds, VPO measurements for *c* > 100 mM were hampered by the formation of films at the surface of solution droplets, which gradually decreased the instrument response.

**Figure 2:**
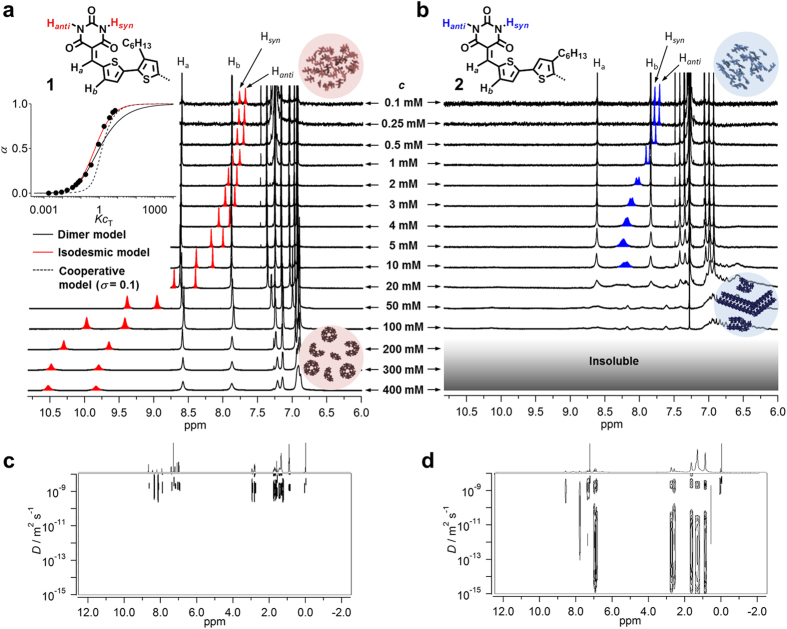
NMR studies of 1 and 2. (**a**) Concentration-dependent ^1^H NMR spectra of **1** (*c* = 0.1–400 mM) in CDCl_3_ at 25 °C. Inset: fraction of aggregated molecules (*α*) calculated from the chemical shift changes of the NH protons of **1** as a function of *Kc*_T_ (*c*_T:_ total concentration of the molecules). Black solid, red solid and black dashed curves represent simulated curves according to dimer, isodesmic, and cooperative (*σ* = *K*_2_/*K* = 0.1) models, respectively. (**b**) Concentration-dependent ^1^H NMR spectra of **2** (*c* = 0.1–100 mM) in CDCl_3_ at 25 °C. (**c**,**d**) DOSY NMR spectra of **1** (*c* = 10 mM) (**c**) and **2** (*c* = 10 mM) (**d**) in CDCl_3_. *D* = diffusion coefficient.

Concentration-dependent ^1^H NMR spectra of CDCl_3_ solutions of the regioisomers further demonstrate preferential formation of distinct hydrogen-bonded assemblies (Fig. 2a,b). At submillimolar concentrations, both the regioisomers displayed sharp signals in the range of 7.67–7.83 ppm for the NH protons of the barbituric acid moiety (H_*syn*_ and H_*anti*_)^34^, indicating the absence of hydrogen bonding interactions. Upon increasing the concentration to *c* = 1 mM, these two NH signals experienced a gradual downfield shift. For **1**, the downfield shift continued upon increasing the concentration up to *c* = 400 mM, and the difference in chemical shift for the two NH signals increased with increasing concentration (Fig. 2a). In contrast, the signals of the aromatic protons H_*a*_ and H_*b*_ remained, while broadening, almost constant as a function of increasing concentration, suggesting that **1** forms hydrogen-bonded oligomeric species in CDCl_3_. The observed large difference between the chemical shifts of H_*syn*_ and H_*anti*_ at *c* > 200 mM (*e.g.* ∆*δ* = 0.69 ppm at *c* = 400 mM) relative to the monomeric state (∆*δ* = 0.08 ppm at *c* = 0.1 mM) illustrates that these protons are subjected to distinct deshielding effects from neighboring molecules. This finding demonstrates that hydrogen-bonding between **1** molecules occurs with a specific configuration. Hence, the formation of screw-shaped hydrogen-bonded rosettes, which is confirmed by the following STM study, and the corresponding intermediate oligomers (*c* ≪ 400 mM) are strongly suggested. Plotting the mole fraction of aggregated molecules *α*, obtained from *α* = [*δ* − *δ*(0.1 mM)]/[*δ*(400 mM) − *δ*(0.1 mM)], against the concentration revealed a sigmoidal transition from the monomeric to the aggregated state. This transition could be fitted well using the equal *K* (isodesmic) model^35^, but deviated substantially from both the dimer model^36^ as well as the cooperative model^37^,^38^ (inset, Fig. 2a). The apparent isodesmic association constant (*K*_iso_) in CDCl_3_ was estimated as *K*_iso_ = 18 M^−1^. The variable-temperature ^1^H NMR measurements (20–60 °C) at *c* = 20 mM showed a change of *α* (0.34–0.14) as a function of temperature, which can be fitted with the isodesmic model with an apparent association constant (*K*_iso_) of 15 M^−1^ (see Supplementary Fig. S13a)^37^. Assuming the formation of hexameric rosettes according to the isodesmic process given in the Supplementary Scheme S1 furnished the *K*_rosette_ (=*K*_iso_^5^) =1.89 × 10^6^ M^−5^ based on the concentration-dependent data^39^.

Compound **2** exhibited a decidedly different behavior in such a concentration-dependent ^1^H NMR study (Fig. 2b). The signals of H_*syn*_ and H_*anti*_ shifted downfield up to *c* = 1 mM, but with diminishing the difference between the two (∆*δ* = 0.07 ppm at *c* = 0.1 mM, ∆*δ* = 0.05 ppm at *c* = 1 mM). This trend is very contrastive to that of **1**, and already at this concentration, different oligomeric species seem to be formed from **2**. Thus, it is evident that the effective molarity (EM) of **2** in hydrogen-bond-directed macrocyclization is very low ([**2**]_EM_ ≈ 1 mM) compared to that of **1** ([**1**]_EM_  ≫ 100 mM). With further increasing concentration, the downfield shift of the two NH signals leveled off at approximately *c* = 4 mM, where the two signals coalesced. This change is very contrastive to that observed for **1**, and implies that hydrogen-bonding between **2** molecules occurs in non-specific configuration even at this concentration. Further increasing the concentration (*c* > 10 mM) caused broadening of all sharp signals with emergence of new complex signals at 6.0–7.0 ppm. These signal patterns unequivocally indicate absence of specific supramolecular species, and reflect the coexistence of diverse polymeric assemblies, the interconversion of which is too slow on the NMR time scale to produce average signals. In a variable-temperature measurement (20–60 °C) at *c* = 20 mM, only the sharpening of proton signals was induced by the disruption of polymeric assemblies (see Supplementary Fig. S13b). All of these observations corroborate the formation of open-ended polydispersed polymeric assemblies.

Diffusion-ordered spectroscopy (DOSY) NMR experiments further provided strong evidence for the formation of different assembly types for **1** and **2** in CDCl_3_. At *c* = 1 mM, similar average diffusion coefficients were observed for both compounds (**1**: *D*_ave_ = 1.32 × 10^−9^ m^2^ s^−1^, **2**: 1.47 × 10^−9^ m^2^ s^−1^) (see Supplementary Fig. S14). These values are comparable with those of non-aggregative *N,N’*-dimethyl derivatives **1-Me** (1.48 × 10^−9^ m^2^ s^−1^) and **2-Me** (1.75 × 10^−9^ m^2^ s^−1^) measured at *c* = 10 mM (see Supplementary Fig. S15). Upon increasing the concentration to *c* = 10 mM, the *D*_ave_ value for **1** changed only marginally (1.32 × 10^−9^ m^2^ s^−1^; Fig. 2c), due to the presence of a low fraction of aggregated molecules (*α* = 0.23; see Supplementary Fig. S12) at this concentration. Unfortunately, DOSY measurements at higher concentrations (*c* > 100 mM; *α* = 0.81) were hampered by severe broadening of the DOSY signals, most likely due to the high concentrations of the solute (5.9 wt%). In striking contrast, a solution of **2** at *c* = 10 mM provided broadly distributed diffusion coefficients (*D* = 4 × 10^−11^–1 × 10^−15^ m^2^ s^−1^), resulting in a *D*_ave_ value of 4.2 × 10^−13^ m^2^ s^−1^ (Fig. 2d), which suggests the presence of large polydispersed assemblies in addition to monomers. Although the exact aggregation number could not be estimated for **1**, the contrastive DOSY NMR results clearly demonstrate distinct inherent aggregation propensity of the two regioisomers.

### Self-Assembly in a 2D-confined space

**Figure 3:**
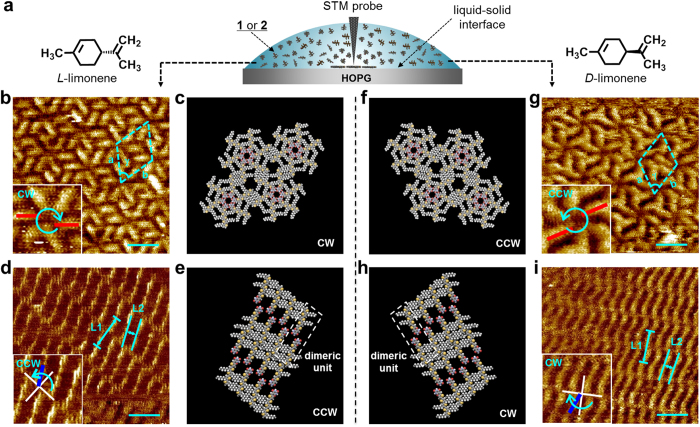
STM studies of 1 and 2. (**a**) Schematic diagram of STM measurements on chiral liquid-solid interface. (**b**,**d**) STM images of **1** (**b**) and **2** (**d**) at the (*S*)-limonene–HOPG interface with the following tunneling conditions: *I* = 1.5 pA, *V* = −1000 mV; *I* = 2.0 pA, *V* = −1000 mV. Concentration of solution is 0.005 mM for both cases. Scale bar, 4 nm. (**g**,**i**) STM images of **1** (**g**) and **2** (**i**) at (*R*)-limonene–HOPG interface with the following tunneling conditions: *I* = 1.5 pA, *V* = −1000 mV; *I* = 3.0 pA, *V* = −900 mV. Concentration of solution is 0.006 mM for both cases. Scale bar, 4 nm. (**c**,**e**,**f**,**h**) Packing models of CW rosettes of **1** (**c**), CCW tapes of **2** (**e**), CCW rosettes of **1** (**f**) and CW tapes of **2** (**h**).

Given the contrastive results in the solution-state studies, we attempted to directly visualize hydrogen-bonded aggregates at the liquid–solid interface by means of scanning tunneling microscopy (STM). According to the previous observations^20^,^40^,^41^, our hydrogen-bonded assemblies could be chiral at the solid–liquid interface^42^,^43^. The formation of two enantiomeric hydrogen-bonded assemblies from achiral components on achiral surface may decrease packing efficiency, which in turn decrease the resolution of the STM imaging. We thus used (*S*)- and (*R*)-limonene as the chiral liquid phase^44^,^45^ in the STM analysis, with the expectation that enantiomerically enriched supramolecular packing through surface confined process (Fig. 3a)^46^.

Figure 3 displays typical STM images of **1** and **2** at the (*S*)- or (*R*)-limonene–HOPG interface. For **1**, closely packed screw-shaped hexameric rosettes that rotate exclusively clockwise (CW) or counterclockwise (CCW) were observed for (*S*)- or (*R*)-limonene, respectively (Fig. 3b,g). The observed unit cell parameters (*α* = 4.9 ± 0.2 nm, *β* = 4.8 ± 0.2 nm, and *γ* = 62 ± 3°) indicated the formation of a pseudohexagonal lattice, which could be reasonably reproduced by molecular modeling (Fig. 3c,f). In sharp contrast, **2** showed lamellar arrangements of the building blocks (Fig. 3d,i). The results of complementary molecular modeling calculations showed that the interlayer spacing L1 (4.6 nm) and the intermolecular distance L2 (1.4 nm) estimated from STM images agree very well with the longer length of the hydrogen-bonded dimeric unit and the distance between the neighbouring dimeric units, respectively (Fig. 3e,h). Exclusive CCW or CW orientations of the dimeric units with respect to the normal of the lamellae were observed for the (*S*)- and (*R*)-limonene–HOPG interfaces, respectively (inset, Fig. 3d,i)^42^. Thus, **2** exclusively forms infinite hydrogen-bonded tape-like molecular arrays, as observed for a barbituric acid-functionalized merocyanine^47^ and a fullerene derivative^48^. These strong preference of the hydrogen-bonded patterns at the liquid–solid interface is concentration-independent (*c* = 0.005–0.1 mM) under the applied experimental conditions^49^, and also is not observed for the 2D molecular packing of regioisomeric *N,N*’-dimethylated derivatives **1-Me** and **2-Me**, which lack the hydrogen-bonding arising from the barbituric acid moieties (see Supplementary Fig. S16). Hence, it can be concluded that the observed 2D assembly of **1** and **2** is supported by hydrogen-bonding interactions and the two regioisomers favor entirely different hydrogen-bonded molecular arrays not only solution phase but also at the solid–liquid interface.

### Mechanism

The preferential formation of the specific hydrogen-bonded motif of **1** and **2** in the 2D-confined space is partially influenced by the kinetic stability of the 2D-packed hydrogen-bonded molecular arrays^50^. However, upon considering the obtained VPO and NMR results, it can be concluded that aggregation driven by such specific hydrogen-bonding motif occur under equilibrium conditions as a result of their thermodynamic preference arising from the regioisomerically attached side chains. In melamine and barbiturate/cyanurate ditopic bicomponent systems, the mechanism for the structural preference between cyclic rosettes and open-ended polymeric motifs has been the subject of controversial discussions^29^,^30^,^51^. On the basis of intricate theoretical simulations for the equilibrium between cyclic and other open-ended oligomers in the bicomponent mixtures, Reinhoudt and coworkers concluded that the steric hindrance, which only affects the internal energy of the open-ended assemblies, should hardly affect the cyclic rosette/open-ended oligomers ratio^30^. This behavior should be attributed to the buffering effect of repulsive interactions in a large number of open-ended species - a notion that was supported by the observation of broad signals in the ^1^H NMR spectra of **2** for *c* > 10 mM. In contrast, they proposed on the basis of a molecular modeling study, that the cyclic rosette/open-ended oligomers ratio should be very sensitive to steric parameters, which directly affect the internal energy of the rosettes.

**Figure 4:**
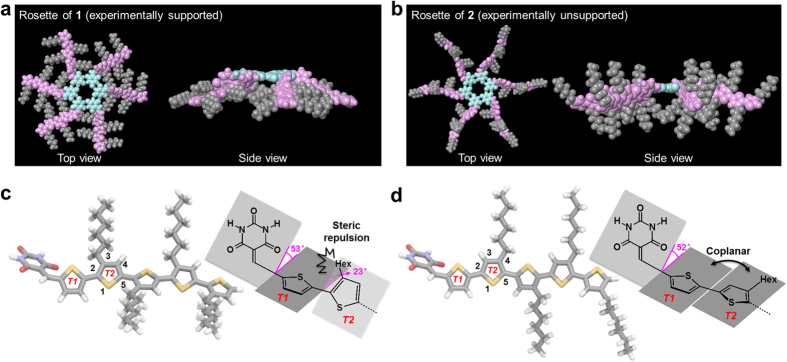
Molecular modeling of 1 and 2. (**a**–**d**) Geometry-optimized structures of hexameric rosettes (**a**,**b**) and monomers (**c**,**d**) of **1** (**a**,**c**) and **2** (**b**,**d**). The molecular modeled structures are shown in vertical (left) and perpendicular (right) direction with respect to the rosette plane. Barbituric acid, oligothiophene, and alkyl chain moieties are colored in light blue, purple, and gray, respectively. The twisting between the barbituric acid, *T1*, and *T2* planes in the monomer structures is shown by a schematic cartoon.

Considering these results, we carried out molecular mechanics calculations on the rosettes as well as on monomers **1** and **2** (Supprementary Methods). Although the energy minimization did not show an appreciable difference between the stabilization energy of the regioisomers upon rosette formation, we observed a significant structural difference between these rosettes (Fig. 4a,b). In rosettes of **1**, all hexyl chains are arranged in the rosette plane, whereas in rosettes of **2**, they are aligned in perpendicular direction with respect to the rosette plane. This structural difference may facilitate van der Waals interactions between neighboring molecules within rosettes and their oligomeric intermediates of **1**, and thus induce stronger thermodynamic preference toward rosette formation for **1** compared to **2**. In the absence of such interactions, the formation of competing tape-like hydrogen-bonded species might prevail as experimentally observed for **2**.

The energy minimization of monomeric **1** and **2** furthermore revealed that the specific orientation of the hexyl chains in the rosettes is an intrinsic structural property of the monomers. In both regioisomers, the barbituric acid plane and the neighboring thiophene plane (*T1*) comprise a dihedral angle of 52–53°, which arises from the steric repulsion between one of the C=O groups of the barbituric acid and *T1* (Fig. 4c,d). In **1**, the adjacent thiophene plane (*T2*) is further twisted, including a dihedral angle of 23° between *T1* and *T2*, which arises from the steric demand of the 3-hexyl group of *T2* in order to counterbalance the twisting between the barbituric acid and *T1*. In contrast, the *T1* and *T2* planes in **2** are almost coplanar (dihedral angle = 0.8°) due to the absence of a hexyl group at the 3-position of *T2*. Hence, it is the twisting between the barbituric acid and *T1* that is predominantly responsible for the molecular shape of **2** observed in its rosette architecture. Accordingly, this subtle conformational difference in the molecular structures, induced by the position of the side chains, should control the initial self-assembly pathways via directional hydrogen bonds.

### Self-organization through solution processing

**Figure 5:**
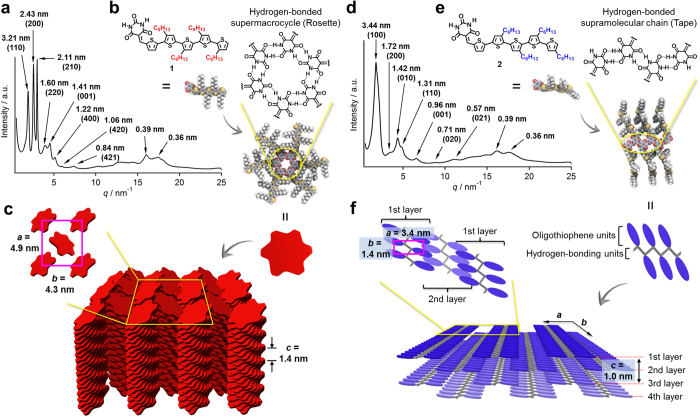
Self-organized structures of 1 and 2 in the bulk. (**a**,**d**) PXRD patterns of bulk samples of **1** (**a**) and **2** (**d**) at 25 °C in a glass capillary (diameter: 1.0 mm). Values in parenthesis denote Miller indices. (**b**,**e**) Molecular models of rosettes of **1** (**b**) and tapes of **2** (**e**). (**c**,**f**) Schematic representations of a proposed packing structures of **1** (**c**) and **2** (**f**) with lattice parameters. In (**c**), only left-handed helical columns are used to show the packing structure. As **1** does not contain a chiral center, both left- and right-handed helical columns should be formed.

The self-organized structures of **1** and **2** via the formation of the previously discussed hydrogen-bonded assemblies were investigated by powder X-ray diffraction (PXRD) measurements on bulk samples of **1** and **2**, which were obtained from solvent evaporation of their CHCl_3_ solutions (*c* = 5 mM). The bulk sample of **1** showed explicit diffraction peaks with *d*-spacings which can be assigned to the diffractions from the (110), (200), (210), (220), (001), (400), (420), and (421) planes of a 3D lattice (Fig. 5a). These results suggest that cyclic rosettes of **1** form a rectangular 2D lattice (space group: *P*2_1_/*a*, lattice parameters: *a* = 4.9 nm, *b* = 4.3 nm) in the *ab* plane, which is complemented by the formation of one-dimensional columns along the *c* axis, obtained from the stacking of these rosettes with a periodic length of 1.4 nm (Fig. 5b,c)^52^,^53^,^54^,^55^. This structural periodicity might correspond to a helical pitch arising from helical stacking of the rosettes. Several diffraction peaks that could be attributed to the columnarly stacked molecules were observed in the wide-angle region (*q* > 15 nm^−1^).

The PXRD pattern of the bulk sample of **2** displayed seven peaks in the small-angle region (Fig. 5d), which could not be indexed considering usual columnar assembling motifs. Here, two sets of diffraction peaks (*d*-spacing of 3.44 and 1.72, as well as 1.42 and 0.71 nm) represent two different structural elements of periodicity (3.4 and 1.4 nm). Considering the presence of an additional diffraction peak with a *d*-spacing of 1.31 nm, these five peaks should be assigned, in order of decreasing *d*-spacing, to the diffractions from the (100), (200), (010), (020), and (110) planes of a 2D rectangular lattice (space group: *P*2*m*, lattice parameters: *a* = 3.4 nm, *b* = 1.4 nm). Given that the diffraction peak with a *d*-spacing of 0.96 nm should be attributed to a structural periodicity element along the *c* axis, the peak with a *d*-spacing of 0.57 nm should be attributed to the diffraction from the (021) plane. In light of the result from the aforementioned STM study, we would like to propose the following structure for self-organized **2** in the bulk. The lattice parameter *b* (1.4 nm) could feasibly be ascribed to the intermolecular distance L2 between dimeric units (Fig. 3d), suggesting that tape-like hydrogen-bonded supramolecular chains form along the *b*-axis (Fig. 5f). Conversely, the lattice parameter *a* (3.4 nm) is shorter than the width L1 (4.6 nm) of the supramolecular chains (Fig. 3d). Presumably, the supramolecular chains are packed in a slipped brick-like motif, where the oligothiophene units interdigitate each other upon stacking in direction of the *c*-axis (Fig. 5e,f). Similar to the case of **1**, several diffraction peaks were observed in the wide-angle region (*q* > 15 nm^−1^), most likely arising from stacked oligothiophene units.

### Co-organization with fullerene semiconductor

The distinct self-assembly pathways of **1** and **2** evolving through solvent evaporation were also manifested in a critical difference regarding their bulk-heterojunction (BHJ) structures with the ball-shaped molecular semiconductor [6,6]-phenyl-C_61_-butyric acid methyl ester (PC_61_BM)^56^,^57^. To obtain insight into the phase separation in BHJ structures, we studied thin films of individual semiconductors as well as their 1:1 (w/w) blends with PC_61_BM (denoted as **1**:PC_61_BM and **2**:PC_61_BM), which are prepared by spin-coating respective homogeneous CHCl_3_ solutions. In differential scanning calorimetry (DSC), **1** exhibited on heating a sharp endothermic peak at 163 °C with an enthalpy change (∆*H*) of 24.3 kJ/mol, which was ascribed to a melting transition (see Supplementary Fig. S17a, broken curve). In contrast, the thermogram of **1**:PC_61_BM displayed during the first heating scan merely a relatively undefined endothermic feature in the range of 107–150 °C (∆*H* = 14.9 kJ/mol) (see Supplementary Fig. S17a, solid curve). The apparent significant discrepancy between the phase transitions of **1** and **1**:PC_61_BM implies that PC_61_BM hampers the self-organization of **1** upon evaporation of the solvent. Contrastingly, the DSC thermograms of **2** and **2**:PC_61_BM did not show such a significant difference, and both showed a sharp endothermic peak around 170 °C ascribed to a melting transition (see Supplementary Fig. S17b). We assume that the negligible impact of PC_61_BM on the organization of **2** reflect the occurrence of a macroscopic phase separation due to a morphological mismatch between the assemblies of **2** and PC_61_BM.

**Figure 6:**
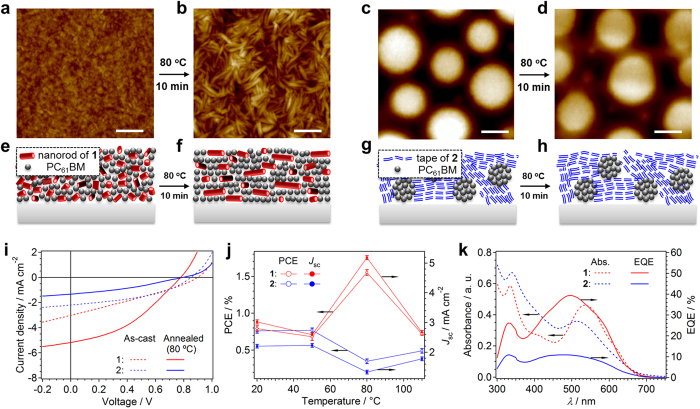
Morphology and photovoltaic properties of p–n heterojunction structures. (**a**–**d**) AFM images of as-cast (**a**,**c**) and annealed (**b**,**d**) thin films of **1**:PC_61_BM (**a**,**b**) and **2**:PC_61_BM (**c**,**d**); scale bar, 200 nm. Thin film samples were prepared by spin-coating CHCl_3_ solutions of **1**:PC_61_BM and **2**:PC_61_BM (*c*_total_ = 20 mg mL^−1^) onto substrates. Annealing conditions: *T* = 80 °C, *t* = 10 min. (**e**–**h**) Schematic illustration of morphological change of **1**:PC_61_BM (**e**,**f**) and **2**:PC_61_BM (**g**,**h**) upon annealing. (**i**) Current–voltage (*J*–*V*) characteristics of BHJ solar cells using 1:1 (w:w) blend films of **1**:PC_61_BM (red lines) and **2**:PC_61_BM (blue lines) before (dotted lines) and after annealing at 80 °C (solid lines). (**j**) PCE and *J*_sc_ values of devices containing **1** (red) and **2** (blue) as a function of the annealing temperature. (**k**) UV-vis absorption (dashed curves) and EQE spectra (solid curves) of thermally annealed (*T* = 80 °C) 1:1 (w/w) blend films of **1**:PC_61_BM (red) and **2**:PC_61_BM (blue). Film thickness: 100–120 nm. Average values of four cells with standard deviation.

AFM images of the surface of the thin film of **1** showed particulate nanostructures (see Supplementary Fig. S18a). Thermal annealing of this film at 80 °C resulted in a directional elongation of particles up to the length of ~100 nm (see Supplementary Fig. S18b). The grazing incidence X-ray diffraction (GI-XRD) image of the film displayed only a wide diffraction circle with a *d*-spacing of ~3.4 nm (see Supplementary Fig. S18c), corresponding to the diffraction from the (110) plane of the rectangular lattice that was observed in the PXRD analysis of the bulk sample of **1** (Fig. 5a). Thermal annealing merely narrowed the width of this diffraction circle. For the thin film of **1**:PC_61_BM, a granularly nanostructured surface was observed by AFM similarly to that of **1** (Fig. 6a), suggesting the absence of any macroscopic phase separation between the two materials (Fig. 6e). Interestingly, thermal annealing of this blend film at 80 °C resulted in the horizontal growth of well-defined nanorods with a uniform diameter (~20 nm) and a length up to ~200 nm (Fig. 6b). In line with the nanostructure change on the surface, the GI-XRD analysis of the thin film of **1**:PC_61_BM revealed a significant reorganization upon thermal annealing. The as-prepared blend film did not show any diffraction circle (see Supplementary Fig. S19c), corroborating the notion that PC_61_BM hampers the self-organization of **1**. However, the GI-XRD image after the thermal annealing featured a clear diffraction spot with a *d*-spacing of 2.5 nm that corresponds to the diffraction from the (200) plane of the rectangular lattice (Fig. 5a) in the meridional direction (see Supplementary Fig. S19d). Thus, the 2D rectangular lattice composed of rosette columns of **1** developed parallelly to the substrate in the presence of PC_61_BM upon thermal annealing, where the *a*-axis (Fig. 5c) is aligned vertically with respect to the substrate surface. Combined with the horizontal growth of the nanorods shown by AFM, the elongation of nanorods is driven by the stacking of rosettes along to the *c*-axis (Fig. 6f).

AFM images of a thin film of **2** showed a pronounced striped texture prior to thermal annealing (see Supplementary Fig. S20a), which developed into a clearer finger-print pattern posterior to thermal annealing at 80 °C (see Supplementary Fig. S20b). The pattern revealed a periodicity of ~4.7 nm, which is commensurate with the width of the tape-like assemblies (Fig. 3d). The GI-XRD image of the as-prepared film of **2** displayed only a weak diffraction circle with a *d*-spacing of 3.6 nm (see Supplementary Fig. S20c), which should correspond to the diffraction from the (100) plane of the rectangular lattice observed for **2** in the bulk (Fig. 5d). Consistent with the AFM results, thermal annealing resulted in an increase of the structural order, as evident from the intensified XRD diffraction posterior to annealing (see Supplementary Fig. S20d). However, the extended lamellar organization **2** caused a significant phase-separation upon mixing with PC_61_BM, as shown by AFM images of the **2**:PC_61_BM film that feature submicrometer-sized grains of PC_61_BM (Fig. 6c,g). This phase-separation feature remained present, even after thermal annealing (Fig. 6d,h). As previously discussed, **1** is able to assemble into discrete cyclic rosettes, while **2** tends to form polymeric hydrogen-bonded tapes, which further assemble into an extended 3D structure. We assume that a morphological mismatch between the assemblies of **2** and PC_61_BM might be responsible for the observed macroscopic phase separation, which should render **2** unfavorable for photovoltaic applications.

Reflecting the significant difference in the phase separation, **1**:PC_61_BM and **2**:PC_61_BM show distinct difference in the photovoltaic property of their solution-processed BHJ solar cells. The devices were fabricated using as-cast blend films, as well as that were thermally annealed at 50, 80, and 110 °C. As **1** and **2** have almost identical optical and electrochemical properties (Fig. 6k and see Supplementary Table S1) as well as comparable hole mobilities in the orders of 10^−4^–10^−5^ cm^2^ V^−1^ s^−1^ (see Supplementary Table S2), the photovoltaic performance of their solar cells should depend strongly on their supramolecular structures^58^. The device performance of **1** and **2** exhibited a diametral bell-shaped dependency on the annealing temperature although the devices with as-cast films showed comparable power conversion efficiency (PCE) of 0.8% and short-circuit current density (*J*_sc_) values (Fig. 6i,j). Devices using **1** showed a notable performance increase upon annealing at 80 °C (PCE: 0.8 → 1.5%), while those using **2** showed a substantially diminished performance (PCE: from 0.8 → 0.3%). As the open-circuit voltage values (*V*_oc_) of all devices were observed consistently at 0.8–0.9 V (see Supplementary Table S3), the annealing-dependence of the PCE may be directly associated with the variation of the *J*_sc_ values, which could in turn be correlated with the distinctive difference in the degree of phase separation because of comparable hole mobilities of **1** and **2**. The BHJ films of **1** and **2** annealed at 80 °C showed a large difference in external quantum efficiency (EQE; **1**: 39.4% at 520 nm; **2**: 10.9% at 510 nm), albeit that only a small difference in their absorption band (300–600 nm) was observed (Fig. 6k). This finding revealed a substantially more efficient photoelectron conversion in the BHJ films of **1** relative to those of **2**. As expected, such a distinct device performance was not observed for devices based on the non-aggregating *N,N*’-dimethylated derivatives **1-Me** and **2-Me**. For BHJ films of these non-aggregative compounds, no significant morphology change was observed upon thermal annealing (Supprementary Fig. 21), and the PCE values of the devices did not exceed 1%, even after thermal annealing (see Supplementary Table S4). Similar relation between the phase-separated structure and the device performance as observed between **1** and **2** have been well decumented for P3HT/PC_61_BM systems^59^, wherein degrees of phase-separation can be controlled physically through solution processing. In the present study the phase-separation could be controlled by means of a supramolecular approach that guarantee high-fidelity self-assembly pathways expanding from the molecular to the bulk level.

## Conclusion

Our experimental data demonstrate that even minor structural alterations in self-assembling building blocks are able to fully control their self-assembly pathways on all levels of hierarchy. This high-fidelity control over self-assembly pathways arises from directional hydrogen-bonding interactions, which render rerouting to other self-assembly pathways difficult. The absence of long aliphatic chains, which are widely used to increase the solubility of functional supramolecular building blocks, is an important factor in our molecular design, as their bulk, flexibility, and van der Waals interactions decisively control the stable self-assembled architectures observed under the conditions applied. Instead of long aliphatic chains, we grafted short alkyl chains onto the *π*-conjugated backbone in order to embed them within the resulting assemblies, which should ensure good solubility without decisively impacting the self-assembled architectures. It should be possible to apply a similar molecular design concept to a variety of other functional molecules, and therefore, we believe that our study expands the scope of controlling self-assembly pathways to obtain specific self-assembled materials including several levels of hierarchy.

## Materials and Methods

The PC_61_MB was purchased from Wako Chemicals (Wako Pure Chemical Industries, Ltd.). The rest of chemical reagents were purchased from TCI Chemicals (Tokyo Chemical Industry Co., Ltd.) and used without further purification. Column chromatography was performed using 63–210 μm silica gel. The solvents for the preparation of assemblies were all spectral grade and used without further purification. ^1^H and ^13^C NMR spectra were recorded on Bruker DPS300 and JEOL JNM-ECA500 NMR spectrometers and chemical shifts are reported in ppm (*δ*) with the signal of TMS as internal standard. ESI-MS spectra were measured on an Exactive (Thermo Scientific). UV/Vis spectra were recorded on a JASCO V660 spectrophotometer. Differential scanning calorimetry was performed on SII DSC6220. Molecular mechanics calculations were performed on MacroModel version 10.4 using AMBER* force field.

### Vapor pressure osmometry (VPO)

Vapor pressure osmomety analysis was performed using KNAUER Vapor Pressure Osmometer K-7000 with EuroOsmo 7000 software. CHCl_3_ was used as solvent for measurements and the temperature for measurements was 25 °C. Benzil was used as a standard for calibration. Sample and standard solutions were prepared by dissolving each compound in CHCl_3_ to achieve a target concentration of 10, 20, 50 or 100 mM. Before performing all sample measurements, the reference condition was established by attaching pure CHCl_3_ on both thermistors and performing AUTOZERO. Solutions of the standard and samples at identical concentrations were entered for every single run and at least three osmograms were recorded for a given concentration. Droplets of pure CHCl_3_ and sample solutions were placed on separate thermistors surrounded by pure solvent vapor. Thermistors were washed after every measurement in order to avoid the contamination by solutions of different concentrations. The size of each drop was kept as constant as possible and equal on both thermistors. In order to keep saturated atmosphere around the thermistors, the chamber was equipped with specially shaped filter papers. The difference in vapor pressures between the two droplets was detected as the difference in temperature at each thermistor. The calibration curve was prepared by measuring CHCl_3_ solutions of benzil. EuroOsmo software was used to draw the graphs and to calculate the calibration constant and molecular weights. Because VPO apparatus is a sensitive piece of equipment, measurement of each concentration was repeated four times to confirm the accuracy of the recorded data.

### DOSY experiments

Sample solutions were prepared by dissolving each compound in CDCl_3_ to achieve a target concentration of 1, 5, 10, 20 or 100 mM. All the solutions were warmed at 60 °C for 10 min in a vial to ensure complete dissolution of the compounds, and equilibrated at room temperature at least 30 min before the measurement. Each solution was transferred to a micro NMR tube with the glass magnetic susceptibility matched to chloroform (Shigemi, BMS-005J). The solution height was adjusted to be 10 mm in the 5 mm O.D. tube. The spectra were recorded on a JEOL JNM-ECX400 spectrometer at the resonance frequency of 399.7822 MHz for ^1^H. The temperature was regulated at 25 °C and no spin was applied during measurements. All the experiments were performed using the bipolar pulse longitudinal eddy current delay (BPP-LED) sequence with spoil gradients applied during the diffusion and eddy current decay periods. The gradient duration was 1 ms and the diffusion time was within the range of 80–100 ms. The gradient strength was varied at even interval on a log-2 scale from 3 to 270 mT m^−1^ in 14 steps. DOSY spectra were generated by the JEOL’s native program Delta, using the adapted CONTIN algorithm for inverse Laplace transforms to build the diffusion dimension.

### Scanning Tunneling Microscopy (STM)

Each compound was dissolved in 1-phenyloctane, (*S*)- or (*R*)-limonene (*c* < 1 × 10^−4^ M). A drop of these solutions was placed on a freshly cleaved highly ordered pyrolytic graphite (HOPG) to prepare a monolayer at the liquid-solid interface. Low current STM observation was performed using a Nanoscope IIIa system (Digital Instruments, Santa Barbara, CA) at freshly cleaved HOPG/each solvent interface. STM tips were mechanically cut and sharpened from Pt/Ir wire (90/10). The STM imaging conditions (current and bias voltage) are given in each figure caption. All the STM images were recorded in the constant current mode, and analyzed by the SPIP software (Image Metrology, Denmark).

### Synchrotron radiation X-ray diffraction experiments

Powder X-ray diffraction (PXRD) patterns of the bulk samples and grazing incidence X-ray diffraction (GI-XRD) images of the film samples were obtained using the BL45XU beamline at SPring-8 (Hyogo, Japan) equipped with an R-AXIS IV++ (Rigaku) imaging plate area detector or with a Pilatus 3 × 2 M (Dectris) detector. The scattering vector, *q* = 4*π*sin*θ*/*λ*, and the position of incident X-ray beam on the detector were calibrated using several orders of layer reflections from silver behenate (*d* = 58.380 Å), where 2*θ* and *λ* refer to the scattering angle and wavelength of the X-ray beam (1.00 Å), respectively. The sample-to-detector distances for PXRD and GI-XRD measurements were 0.40 and 0.38 m, respectively. The obtained diffraction patterns and images were integrated along the Debye-Scherrer ring to afford one-dimensional intensity data using the FIT2D software (http://www.esrf.eu/computing/scientific/FIT2D/). The lattice parameters were refined using the CellCalc ver. 2.10 software (http://homepage2.nifty.com/~hsc/soft/cellcalc_e.html).

### Atomic force microscopy (AFM)

AFM images were acquired under ambient conditions using Multimode 8 Nanoscope V (Bruker Instruments) in Tapping mode or Peak Force Tapping (Scanasyst) mode. Silicon cantilevers (MPP-21100-10) with a spring constant of 3 N/m and frequency of 70 kHz (nominal value, Bruker, Japan) or silicon cantilevers (SCANASYST-AIR) with a spring constant of 0.4 N/m and frequency of 70 kHz (nominal value, Bruker, Japan) were used for Tapping mode and Scanasyst mode, respectively. Samples were prepared by spin-coating assembly solutions onto a freshly cleaved highly-oriented pyrolytic graphite (HOPG).

### Photovoltaic devices

Bulk heterojunction solar cell devices were fabricated on indium-tin oxide (ITO) coated glass with a device structure Al/Ca/BHJ film/PEDOT:PSS/ITO. The ITO was cleaned with acetone and 2-propanol in ultrasonic bath. The resultant ITO substrates were exposed to UV-ozone for 20 min and then coated with PEDOT:PSS [poly(3,4-ethylene dioxythiophene):poly(styrene sulfonate)] (thickness: ca. 30 nm). The substrates were heated for 20 min at 120 °C to remove residual water. In a N_2_ glove box, 0.5 mL of chloroform solutions containing PC_61_BM (5 mg, Luminescence Technology Corp., Taiwan Province) and donor materials (5 mg) were spin-coated (1000 rpm for 30 sec) onto the substrate. The thickness of the resulting BHJ films (100–120 nm) was determined by using a DEKTAK surface profiler (Bruker AXS). The substrates were then moved to a high-vacuum chamber, and the top electrode was evaporated through a shadow mask (Ca:10 nm, Al:90 nm) to give solar cells with an active area of 0.04 cm^2^. Finally, the devices were encapsulated by a glass lid in the nitrogen glove box system. The *J-V* characteristics of the solar cells were evaluated by using a Keithley 2400 source-measure unit. The AM 1.5 G light was provided by a filtered Xe lamp. The intensity of 100 mW cm^−2^ of the AM 1.5 G light was determined by using a calibrated inorganic solar cell from the National Institute of Advanced Industrial Science and Technology (Japan). No spectral mismatch factor was included in the calculation of the efficiency.

### Hole mobility measurements

Hole mobilities of **1** and **2** films (prepared from 10-mg/ml CHCl_3_ solutions) were estimated by charge-only space-charge limited current (SCLC) method. The SCLC is described by *J* = *9ε*_0_*ε*_r_*μV*^2^*/8L*^3^, where *J* is the current density, *L* is the film thickness of the active layer, *μ* is the hole mobility, *ε*_r_ is the relative dielectric constant of the transport medium, *ε*_0_ is the permittivity of free space (8.85 × 10^−12^ F m^−1^), *V* is the applied voltage to the device. SCLC measurements were carried out with a device structure of ITO/PEDOT:PSS/active layer/MoO_3_/Al by taking the current density in the range 0–10 V and fitting the results to a space-charge limited form.

## Additional Information

**How to cite this article:** Lin, X. *et al*. High-fidelity self-assembly pathways for hydrogen-bonding molecular semiconductors. *Sci. Rep.* **7**, 43098; doi: 10.1038/srep43098 (2017).

**Publisher's note:** Springer Nature remains neutral with regard to jurisdictional claims in published maps and institutional affiliations.

## Supplementary Material

Supplementary Information
