# Supplementary material for: High-fidelity self-assembly pathways for hydrogen-bonding molecular semiconductors
Source: Sci Rep. 2017 Feb 22;7:43098. doi: 10.1038/srep43098 (PMC5320534; doi:10.1038/srep43098)
Supplement: Supplementary Information [file srep43098-s1.pdf]

SUPPLEMENTARY INFORMATION

High-fidelity self-assembly pathways for hydrogen-bonding molecular semiconductors

Xu Lin[1], Mika Suzuki[1], Marina Gushiken[1], Mitsuaki Yamauchi[1], Takashi Karatsu[1], Takahiro Kizaki[2], Yuki Tani[2], Ken-ichi Nakayama[3], Mitsuharu Suzuki[4], Hiroko Yamada[4], Takashi Kajitani[5,6], Takanori Fukushima[5], Yoshihiro Kikkawa[7] and Shiki Yagai[1,*]

[1]Graduate School of Engineering, Chiba University, 1-33 Yayoi-cho, Inage-ku, Chiba 263-8522, Japan. [2]Graduate School of Science and Engineering, Yamagata University, 4-3-16 Jonan, Yonezawa, Yamagata 992-8510, Japan. [3]Graduate School of Engineering, Osaka University, 2-1 Yamadaoka, Suita, Osaka 565-0871, Japan. [4]Graduate School of Material Science, Nara Institute of Science and Technology (NAIST), 8916-5 Takayama-cho, Ikoma, Nara 630-0192, Japan. [5]Laboratory for Chemistry and Life Science, Institute of Innovative Research, Tokyo Institute of Technology, 4259 Nagatsuta, Midori-ku, Yokohama 226-8503, Japan. [6]RIKEN SPring-8 Center, 1-1-1 Kouto, Sayo, Hyogo 679-5148, Japan. [7]National Institute of Advanced Industrial Science and Technology (AIST), 1-1-1 Higashi, Tsukuba, Ibaraki 305-8562, Japan. (Email: yagai@faculty.chiba-u.jp)

Table of Contents

Synthesis and Characterization....................2

Supplementary Data....................13

Supplementary References....................20

Synthesis and Characterization

Barbiturate-conjugated thienyl[oligohexyl(thiophene)]s **2** and non-hydrogen-bonding derivatives **2-Me** were synthesized according to the following steps. Compounds **1**, **1-Me**, **3**, **4**, **5**, **6** and **7** were synthesized according to Scheme S1 and details were reported previously.[S1]

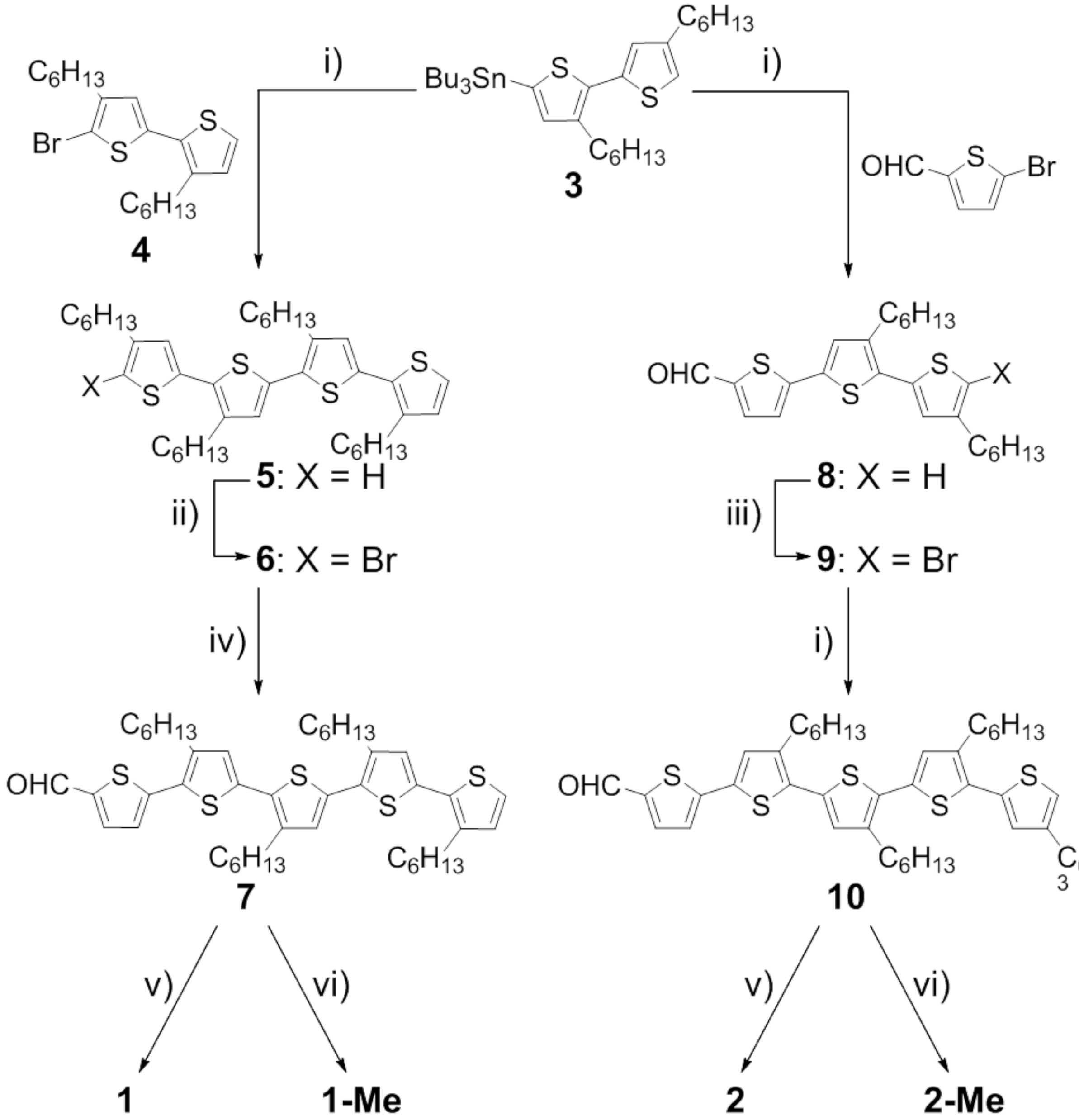

i) $Pd(PPh_3)_4$, DMF, microwave 140 °C; ii) NBS, $CHCl_3$/acetic acid, 0 °C→r.t.; iii) NBS, DMF, 0 °C →r.t.; iv) 5-formylthiophene-2-boronic acid, K_2CO_3, $Pd(dppf)Cl_2$, toluene/MeOH, 80 °C; v) barbituric acid, EtOH, reflux; vi) 1,3-dimethylbarbituric acid, EtOH, reflux.

Synthesis of compound 8: A 5-mL of microwave reactor vessel was charged with 5-bromothiophene-2-carboxaldehyde (100 mg, 0.52 mmol), **3** (486 mg, 0.78 mmol), $Pd(PPh_3)_4$ (20 mg, 0.016 mmol), and DMF (3 mL), and the vessel was placed into a microwave reactor. The reaction temperature and period were set to 140 °C and 7 min, respectively. The reaction mixture was diluted with ethyl acetate, and washed twice with water and once with brine. The organic layer was dried with Na_2SO_4, filtered and evaporated to dryness. The resulting liquid was purified by column chromatography (10 w/w% K_2CO_3-silica gel, eluent: $CHCl_3$/hexane = 2:3 (v/v)) to give pure compound **8** as an orange liquid (227 mg, 98%). ^1H NMR (300 MHz, $CDCl_3$): δ = 9.85 (s, 1H, CH_aO), 7.66 (d, J = 4.1 Hz, 1H, thienyl-H_b), 7.21 (d, J = 3.9 Hz, 1H, thienyl-H_c), 7.18 (s, 1H, thienyl-H_d), 7.00 (s, 1H, thienyl-H_e), 6.94 (s, 1H, thienyl-H_f), 2.74 (t, J = 7.7 Hz, 2H, CH_g), 2.61 (t, J = 7.4 Hz, 2H, CH_h), 1.65–1.54 (m, 4H, CH_i), 1.41–1.32 (m, 12H, CH_j), 0.92–0.87 (m, 6H, CH_k); ^{13}C NMR (75.49 MHz, $CDCl_3$): δ = 182.21, 146.98, 143.79, 141.33, 140.37, 137.36, 134.84, 133.25, 133.03, 128.90, 127.59, 123.78, 120.71, 31.68, 31.61, 30.43, 30.36, 29.31, 29.21, 29.02, 22.63, 22.40, 14.12, 14.09; HRMS (ESI) m/z calcd. for $C_{25}H_{33}OS_3$ 445.1688 $[M+H]^+$; found 445.1674.

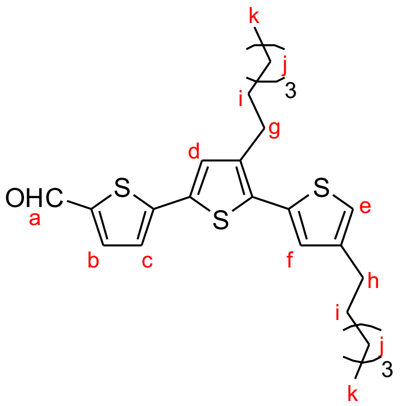

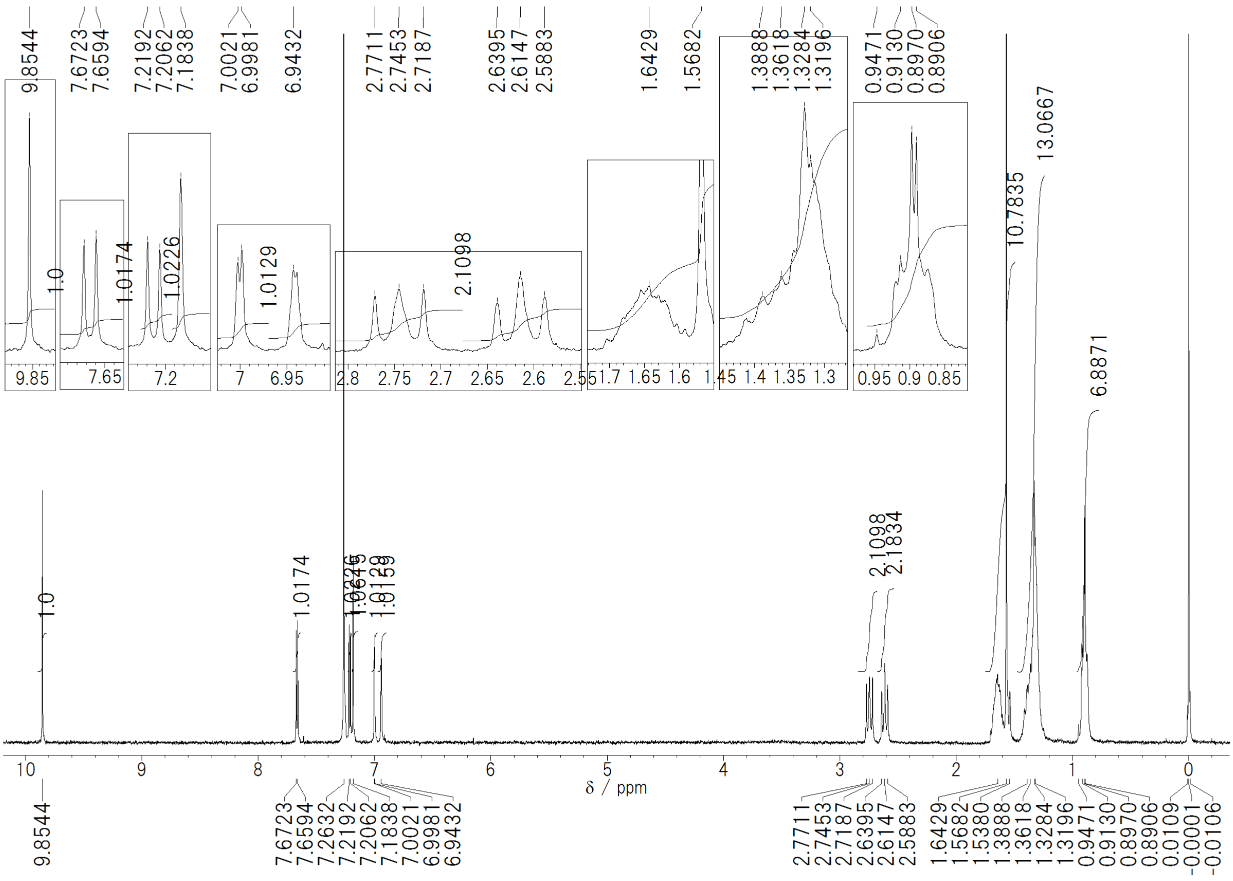

Figure S1. ^{1}H NMR spectrum of **8** in $CDCl_3$ at 293 K.

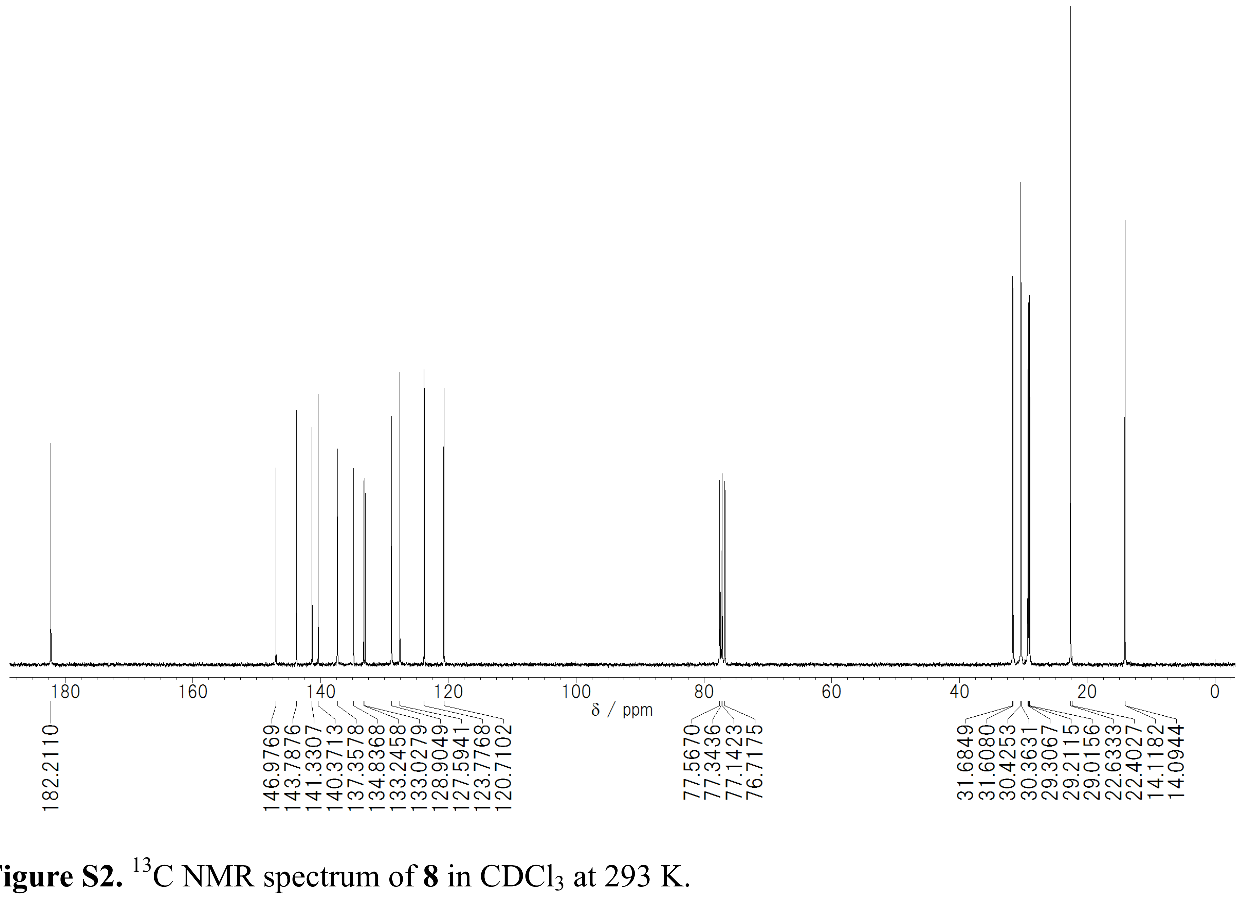

Figure S2. ^{13}C NMR spectrum of **8** in $CDCl_3$ at 293 K.

Synthesis of compound 9: To an ice-cooled DMF solution (10 mL) containing **8** (227 mg, 0.51 mmol), *N*-bromosuccinimide (91 mg, 0.51 mmol) was added in three portions with interval of 10 min. The reaction mixture was warmed to r.t. and stirred overnight. The mixture was diluted with ethyl acetate, and washed twice with water and once with brine. The organic layer was dried over Na_2SO_4, filtered and evaporated to dryness to give compound **9** as an orange liquid in almost quantitative yield without purification (268 mg, 100%). ^{1}H NMR (300 MHz, $CDCl_3$): δ = 9.86 (s, 1H, CH_aO), 7.67 (d, J = 3.9 Hz, 1H, thienyl-H_b), 7.21 (d, J = 4.0 Hz, 1H, thienyl-H_c), 7.17 (s, 1H, thienyl-H_d), 6.85 (s, 1H thienyl-H_e), 2.70 (t, J = 7.8 Hz, 2H, CH_f), 2.57 (t, J = 7.5 Hz, 2H, CH_g), 1.62–1.56 (m, 4H CH_h), 1.33–1.25 (m, 12H, CH_i), 0.92–0.87 (m, 6H CH_j); ^{13}C NMR (75.49 MHz, $CDCl_3$): δ = 182.80, 147.14, 143.07, 141.95, 141.34, 137.85, 135.05, 133.95, 132.49, 129.29, 127.52, 126.77, 124.44, 109.94, 32.02, 30.86, 30.05, 29.92, 29.70, 29.59, 29.33, 23.03, 14.53; HRMS (ESI) m/z calcd. for $C_{25}H_{32}OBrS_3$ 523.0793 $[M+H]^+$; found 523.0783.

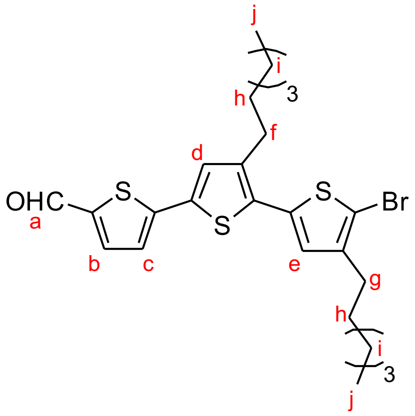

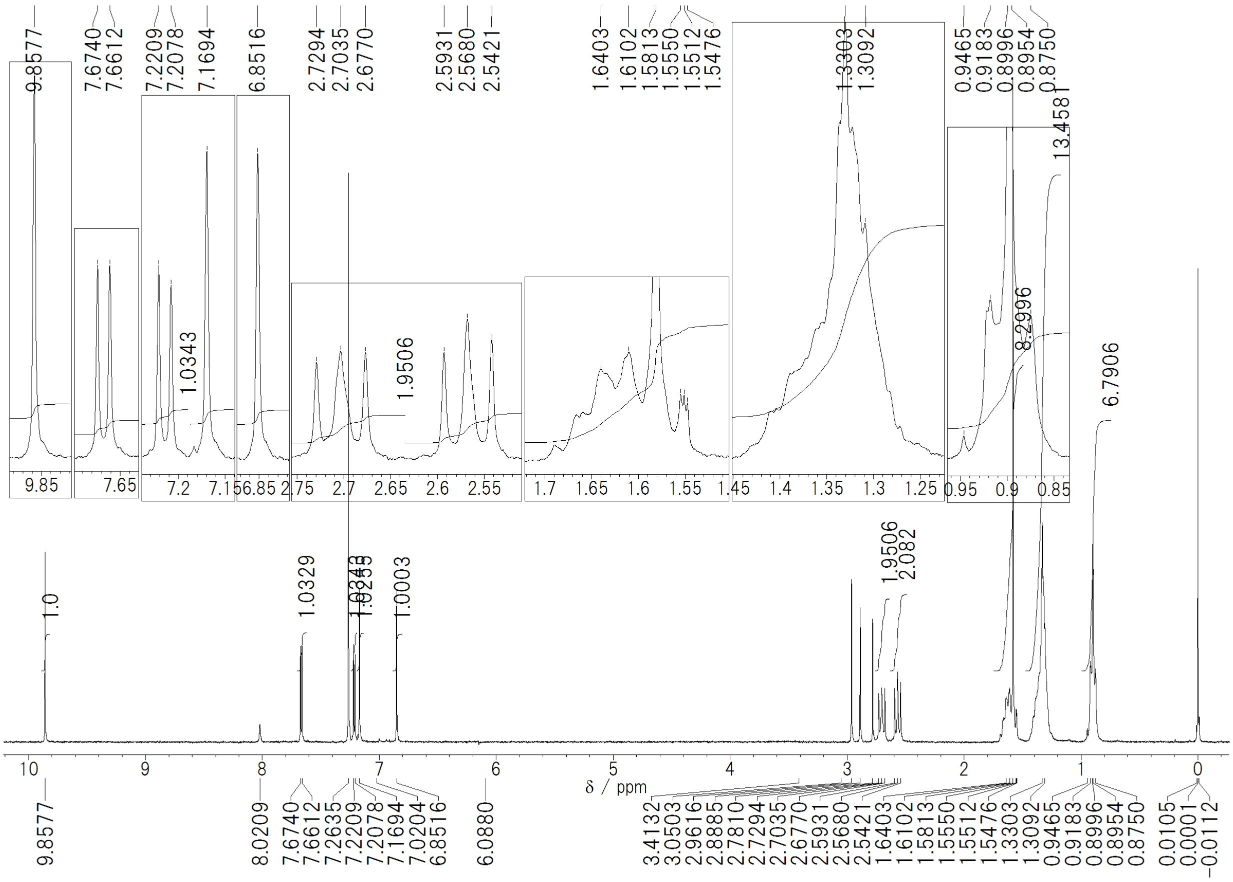

Figure S3. ^{1}H NMR spectrum of **9** in $CDCl_3$ at 293 K.

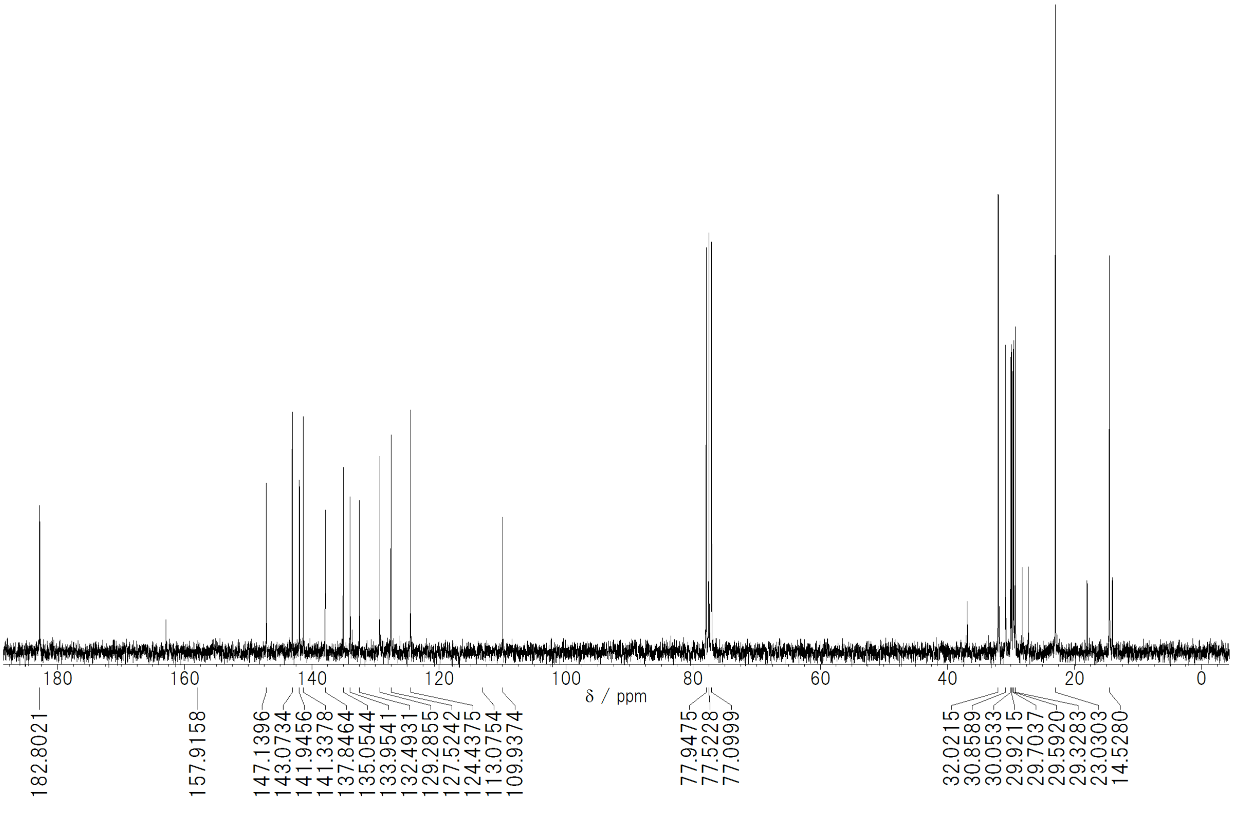

Figure S4. ^{13}C NMR spectrum of **9** in $CDCl_3$ at 293 K.

Synthesis of compound 10: A 5-mL of microwave reactor vessel was charged with **9** (268 mg, 0.51 mmol), **3** (383 mg, 0.61 mmol), $Pd(PPh_3)_4$ (20 mg, 0.016 mmol), and DMF (3 mL), and the vessel was placed into a microwave reactor. The reaction temperature and period was set to 140 °C and 7 min, respectively. The reaction mixture was diluted with ethyl acetate, and washed twice with water and once with brine. The organic layer was dried with Na_2SO_4, filtered and evaporated to dryness. The resulting liquid was purified by column chromatography (10 w/w% K_2CO_3-silica gel, eluent: $CHCl_3$/hexane = 2:3 (v/v)) to give pure compound **10** as an orange liquid (263 mg, 56%).

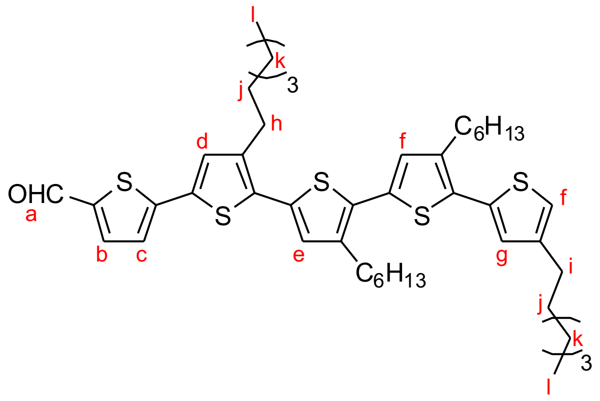

^{1}H NMR (300 MHz, $CDCl_3$): δ = 9.86 (s, 1H, CH_aO), 7.67 (d, J = 3.9 Hz, 1H, thienyl-H_b), 7.22 (d, J = 4.0 Hz, 1H, thienyl-H_c), 7.19 (s, 1H, thienyl-H_d), 7.00 (s, 1H, thienyl-H_e), 6.97 (m, 2H, thienyl-H_f), 6.91 (s, 1H, thienyl-H_g), 2.81–2.73 (m, 6H, CH_h), 2.61 (t, J = 7.7 Hz, 2H, CH_i), 1.68–1.60 (m, 8H, CH_j), 1.42–1.25 (m, 24H, CH_k), 0.90–0.89 (m, 12H, CH_l); ^{13}C NMR (75.49 MHz, $CDCl_3$): δ = 182.83, 147.43, 144.11, 141.81, 141.06, 140.27, 140.05, 135.80, 133.54, 133.26, 133.21, 131.88, 131.74, 129.60, 129.46, 129.06, 128.45, 127.61, 125.72, 124.32, 120.53, 32.11, 32.08, 31.01, 30.93, 30.84, 29.91, 29.84, 29.69, 29.47, 23.08, 14.56; HRMS (ESI) m/z calcd. for $C_{45}H_{61}OS_5$ 777.3320 $[M+H]^+$; found 777.3301.

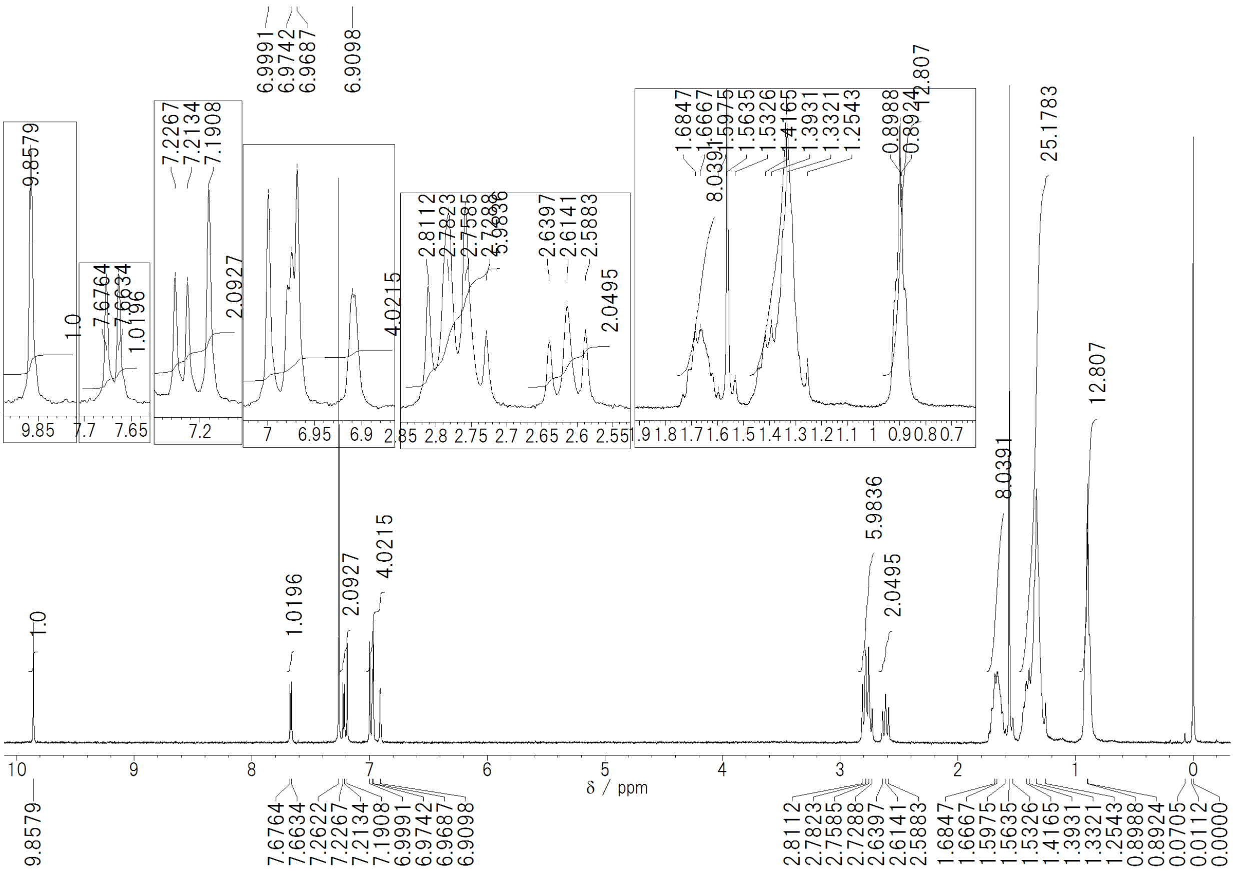

Figure S5. ^{1}H NMR spectrum of **10** in $CDCl_3$ at 293 K.

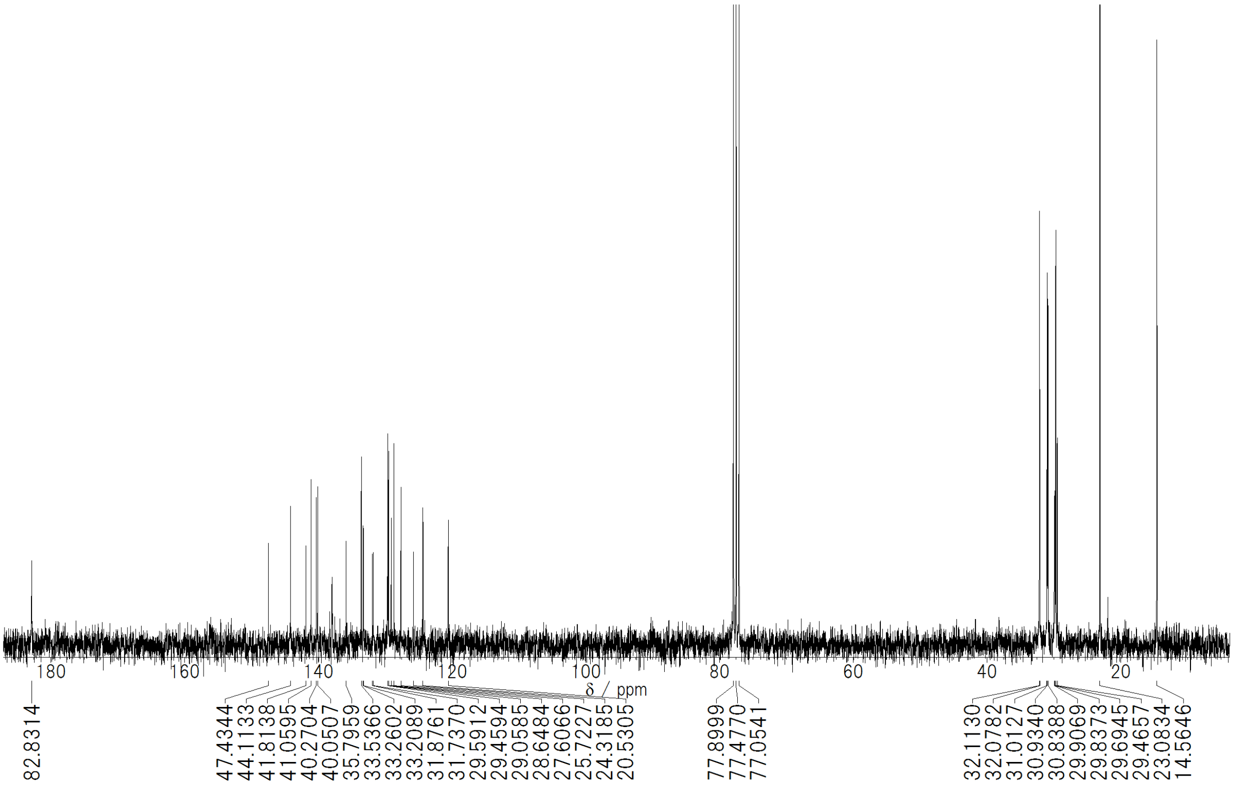

Figure S6. ^{13}C NMR spectrum of **10** in $CDCl_3$ at 293 K.

Synthesis of compound 2: A mixture of **10** (263 mg, 0.34 mmol) and barbituric acid (150 mg, 1.20 mmol) in EtOH (5 mL) was refluxed for 3 h. The reaction mixture was cooled to r.t., and the resulting precipitates were collected by filtration and washed with hot water repeatedly. The residual solid was reprecipitated from a $CHCl_3$–hexane mixture to give nearly pure compound **2** as a black-purple solid (168 mg, 56%). This compound was further purified by preparative GPC (eluent: $CHCl_3$). Mp 157 °C (DSC 2nd heating); ^{1}H NMR (500 MHz, $CDCl_3$, 60 °C): δ = 8.59 (s, 1H, C=CH_a–thienyl), 8.02 (br-s, 1H, NH_b), 7.99 (br-s, 1H, NH_c), 7.82 (d, J = 4.3 Hz, 1H, thienyl-H_d), 7.40 (s, 1H, thienyl-H_e), 7.34 (d, J = 4.2 Hz, 1H, thienyl-H_f), 7.04 (s, 1H, thienyl-H_g), 6.98 (m, 2H, thienyl-H_h), 6.91 (s, 1H, thienyl-H_i), 2.82–2.72 (m, 6H, CH_j), 2.61 (t, J = 7.6 Hz, 2H, CH_k), 1.73–1.62 (m, 8H, CH_l), 1.45–1.28 (m, 24H, CH_m), 0.93–0.86 (m, 12H, CH_n); ^{13}C NMR (125.77 MHz, tetrachloroethane-d_2, 130 °C): δ = 162.72, 162.01, 154.75, 148.51, 148.18, 147.18, 143.83, 141.57, 140.35, 140.01, 135.66, 135.62, 135.14, 133.47, 133.40, 133.02, 132.36, 131.91, 130.51, 129.75, 129.04, 127.64, 124.79, 120.34, 108.14, 31.65, 31.62, 30.72, 30.57, 30.40, 30.36, 30.34, 30.28, 29.66, 29.53, 29.40, 29.17, 29.14, 28.96, 22.51, 13.86; HRMS (ESI) m/z calcd. for $C_{49}H_{63}O_3N_2S_5$ 887.3437 $[M+H]^+$; found 887.3431.

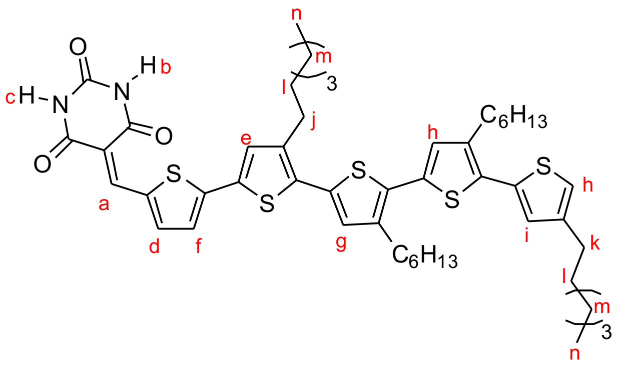

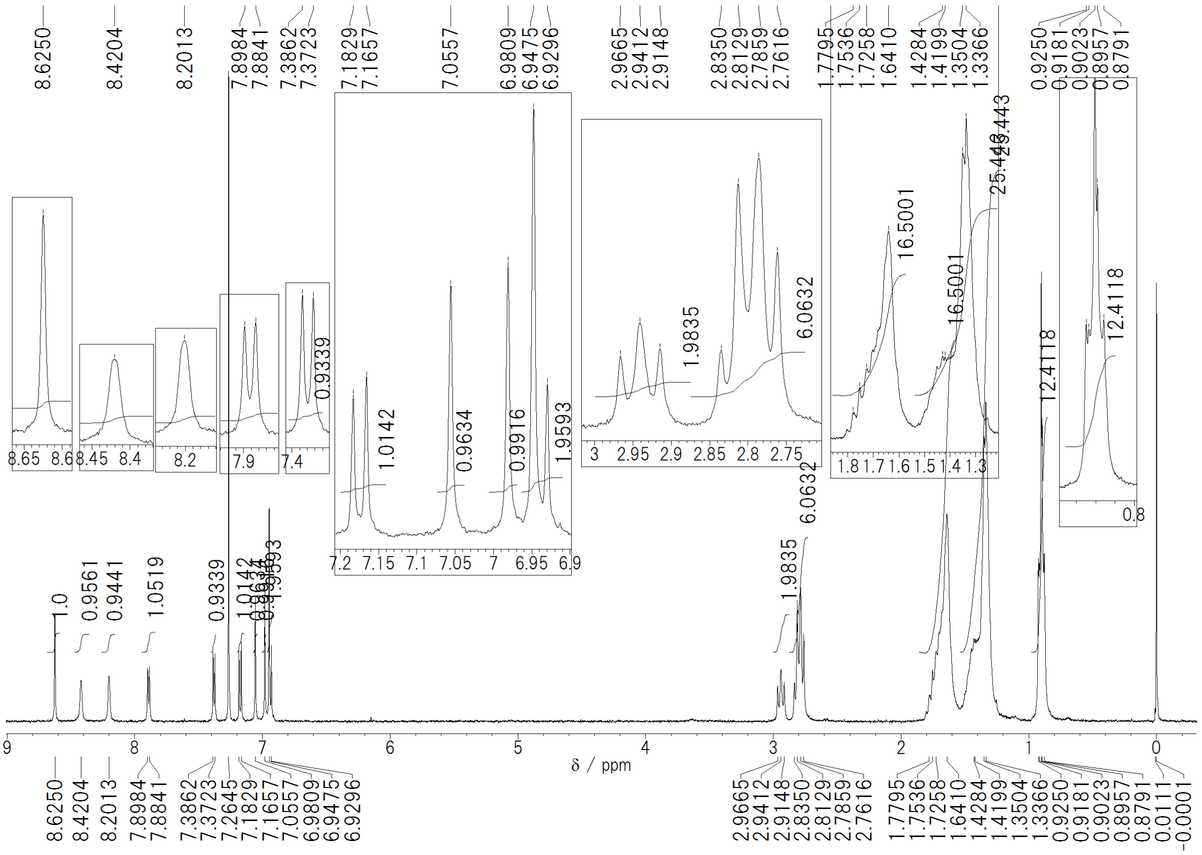

Figure S7. ^{1}H NMR spectrum of **2** in $CDCl_3$ at 293 K.

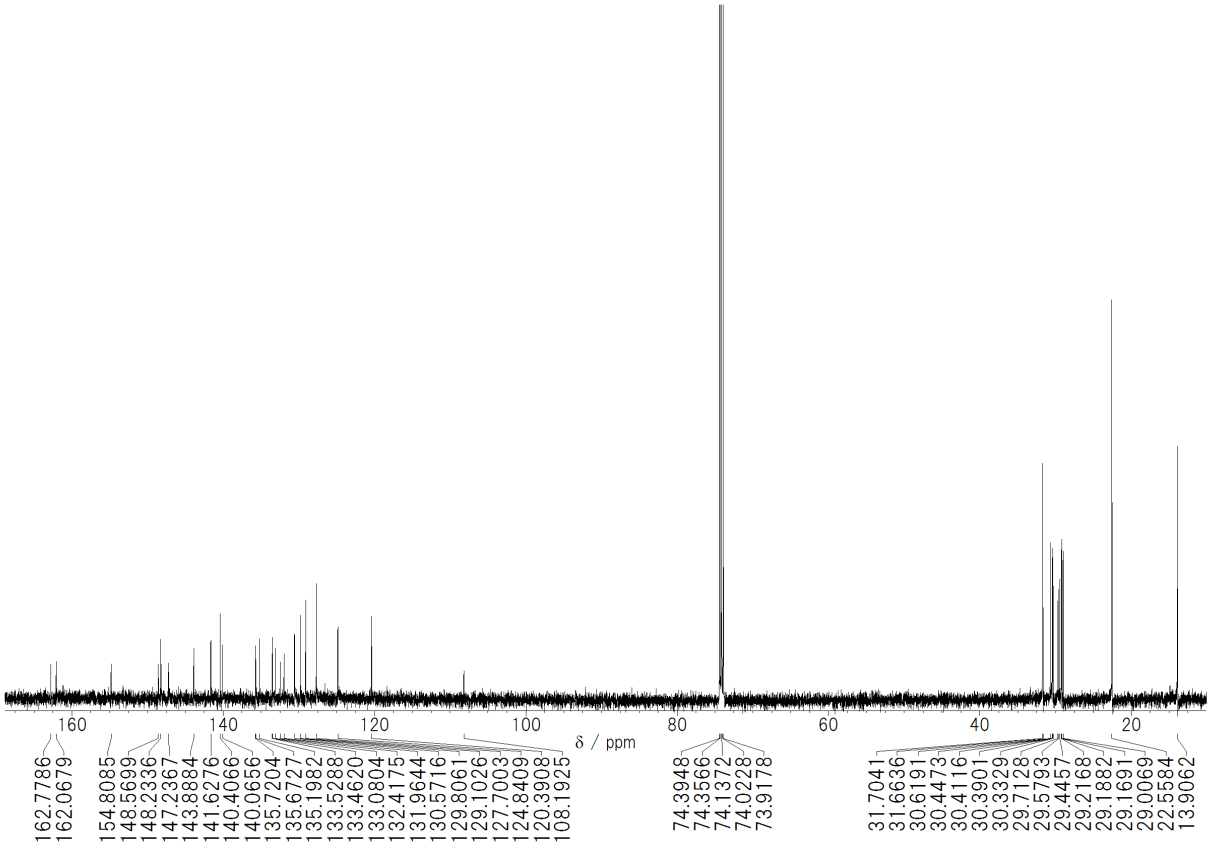

Figure S8. ^{13}C NMR spectrum of **2** in tetrachloroethane-d_2 at 403 K.

Synthesis of compound 2-Me: A mixture of **10** (81 mg, 0.10 mmol) and *N,N'*-dimethylbarbituric acid (70 mg, 0.45 mmol) in EtOH (5 mL) was refluxed for 3 h. The reaction mixture was cooled to r.t., and the resulting precipitates were collected by filtration and washed with hot water repeatedly. The residual solid was reprecipitated from a $CHCl_3$–EtOH mixture to give nearly pure compound **2-Me** as a black-purple solid (89 mg, 97%). This compound was further purified by preparative GPC (eluent: $CHCl_3$). Mp 97 °C (DSC); ^{1}H NMR (500 MHz, $CDCl_3$): δ = 8.63 (s, 1H, C=CH_a–thienyl), 7.78 (d, *J* = 4.4 Hz, 1H, thienyl-H_b), 7.40 (s, 1H, thienyl-H_c), 7.32 (d, *J* = 4.3 Hz, 1H, thienyl-H_d), 7.03 (s, 1H, thienyl-H_e), 6.98 (s, 2H, thienyl-H_f), 6.91 (s, 1H, thienyl-H_g), 3.45 (s, 3H, N–CH_h), 3.43 (s, 3H, N–CH_i), 2.82–2.73 (m, 6H, CH_j), 2.62 (t, *J* = 7.6 Hz, 2H, CH_k), 1.73–1.60 (m, 8H, CH_l), 1.46–1.28 (m, 24H, CH_m), 0.93–0.87 (m, 12H, CH_n); ^{13}C NMR (75.49 MHz, $CDCl_3$): δ = 163.18, 162.56, 153.98, 151.85, 148.59, 147.58, 144.11,141.45, 140.35, 140.08, 135.76, 135.75, 134.69, 133.66, 133.47, 133.17, 132.21, 131.84, 130.60, 129.76, 129.12, 127.64, 124.77, 120.55, 108.95, 32.07, 30.97, 30.90, 30.88, 30.81, 30.78, 29.98, 29.80, 29.67, 29.64, 29.42, 29.29, 28.55, 23.04, 14.52; HRMS (ESI) *m/z* calcd. for $C_{51}H_{67}O_3N_2S_5$ 915.3750 $[M+H]^+$; found 915.3740.

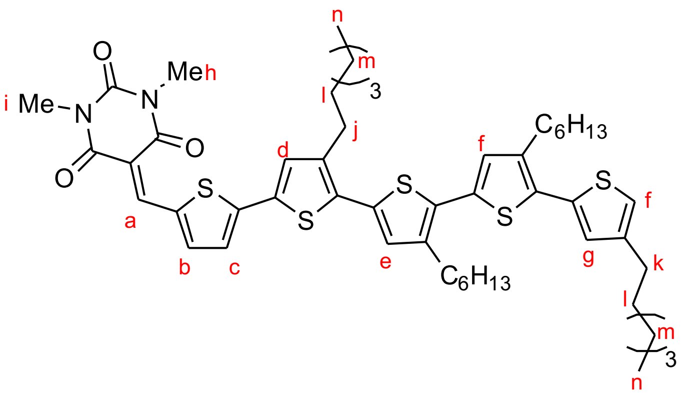

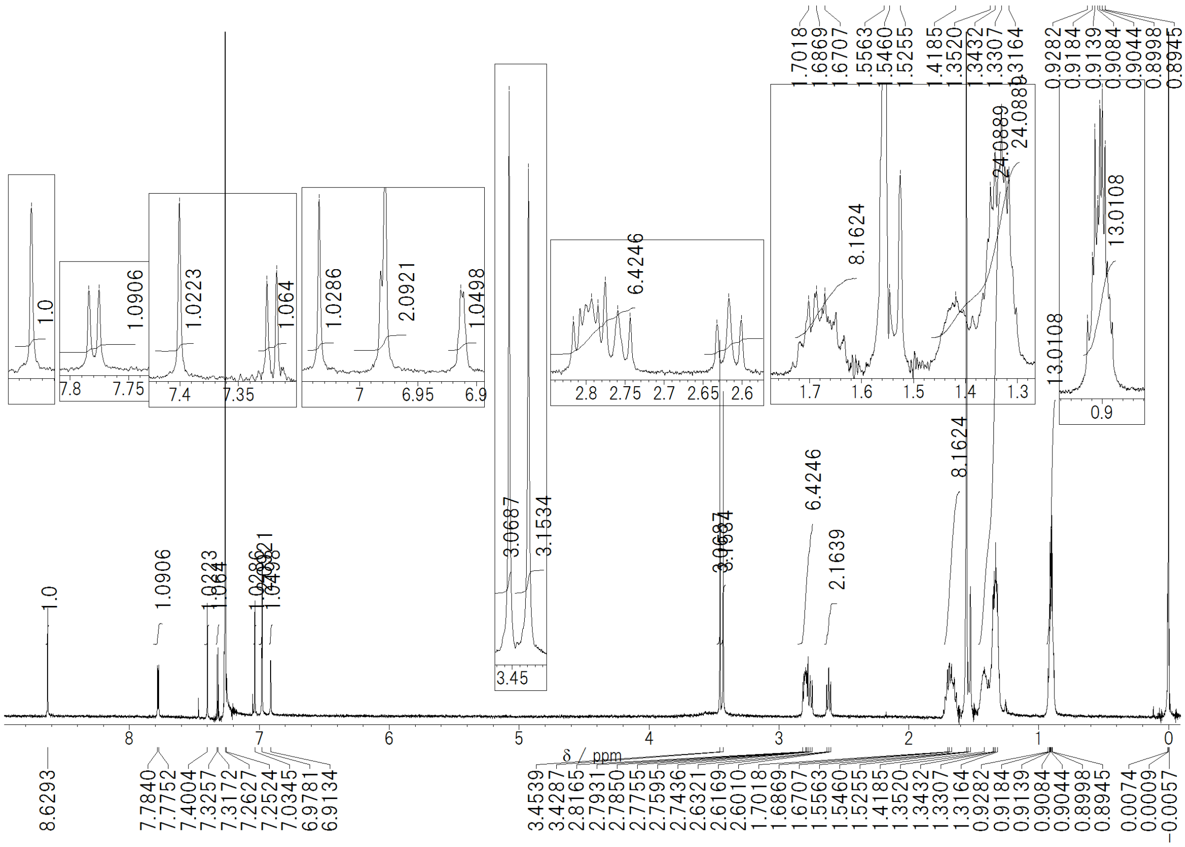

Figure S9. ^{1}H NMR spectrum of **2-Me** in $CDCl_3$ at 293 K.

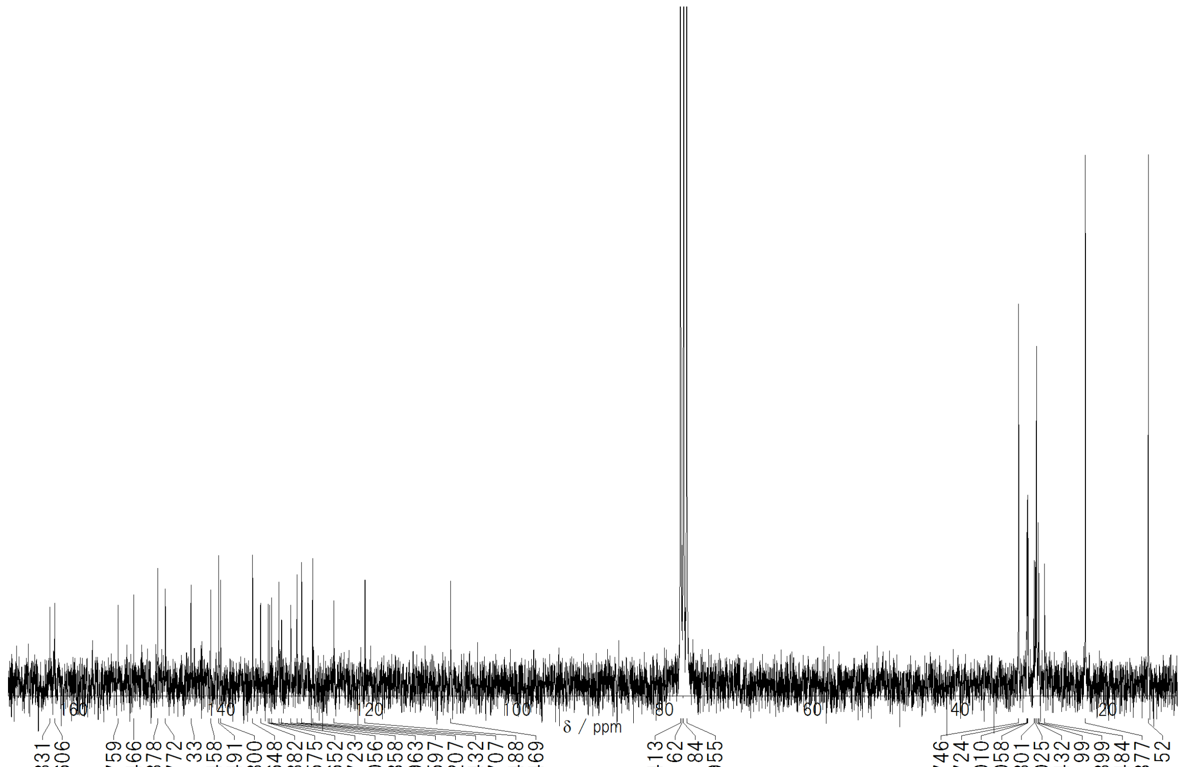

Figure S10. ^{13}C NMR spectrum of **2-Me** in $CDCl_3$ at 293 K.

Supplementary Data

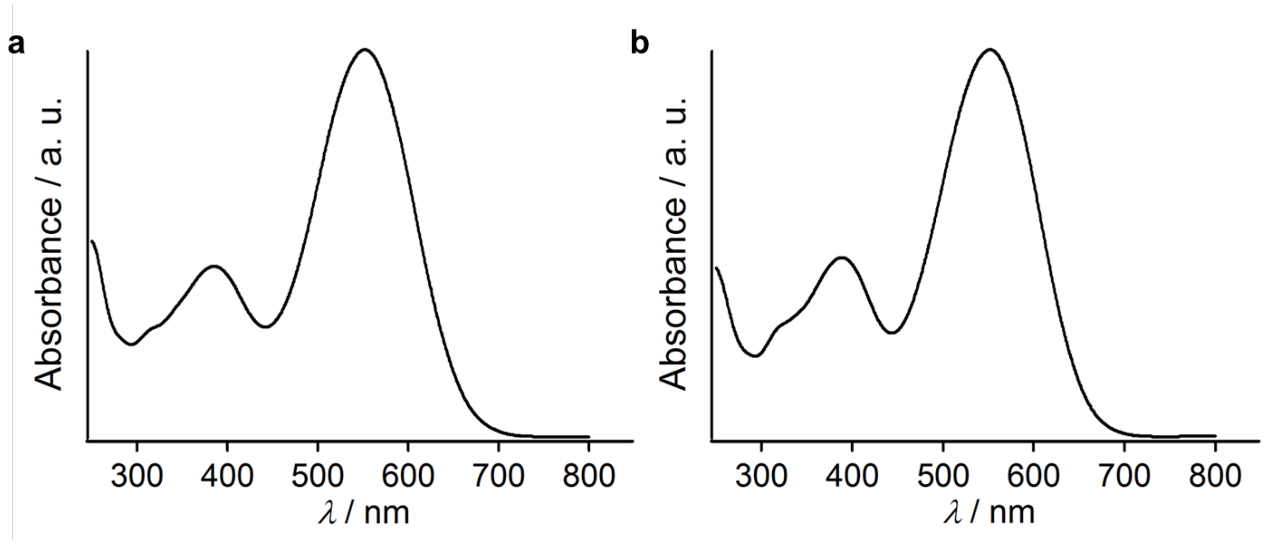

Figure S11. UV-vis absorption spectra of **1**(**a**) and **2**(**b**) (c = 0.01 mM) in $CHCl_3$.

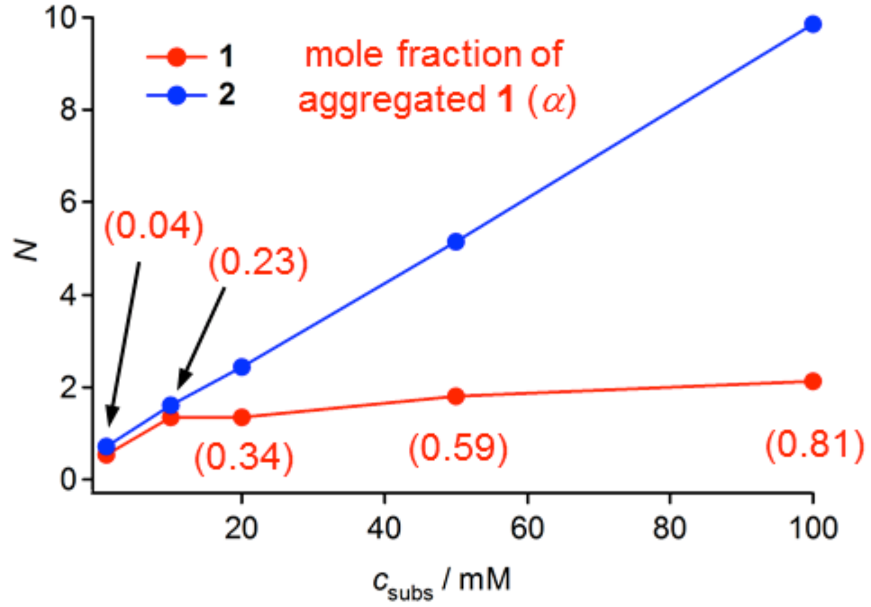

Figure S12. VPO-derived plots of the aggregation number $N = c_{subs}/c_{collig}$ (c_{mon}: 10 – 100 mM) as a function of the monomer concentration of **1** (red) and **2** (blue). Numbers in parentheses indicate the fraction of aggregated molecules (α) of **1**.

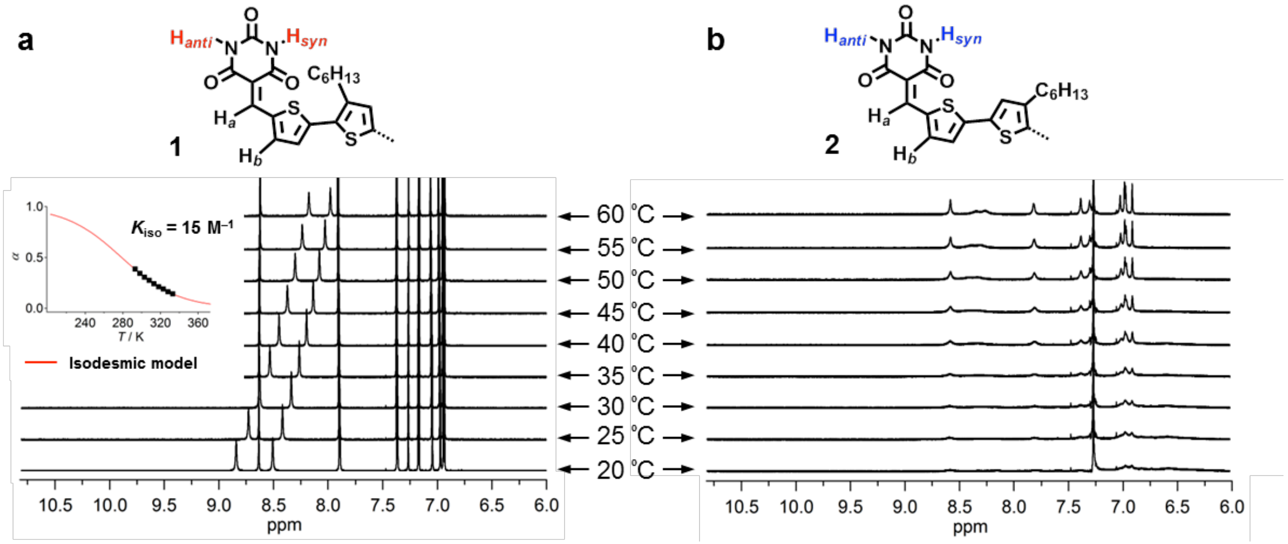

Figure S13. Temperature-dependent ^{1}H NMR spectra of **1** (**a**) and **2** (**b**) at c = 20 mM in $CDCl_3$ upon heating from 20 °C to 60 °C. Inset in (**a**): Inset: fraction of aggregated molecules (α) calculated from the chemical shift changes of the NH protons of **1** as a function of the temperature. Red solid curve represent simulated curves according to isodesmic model.

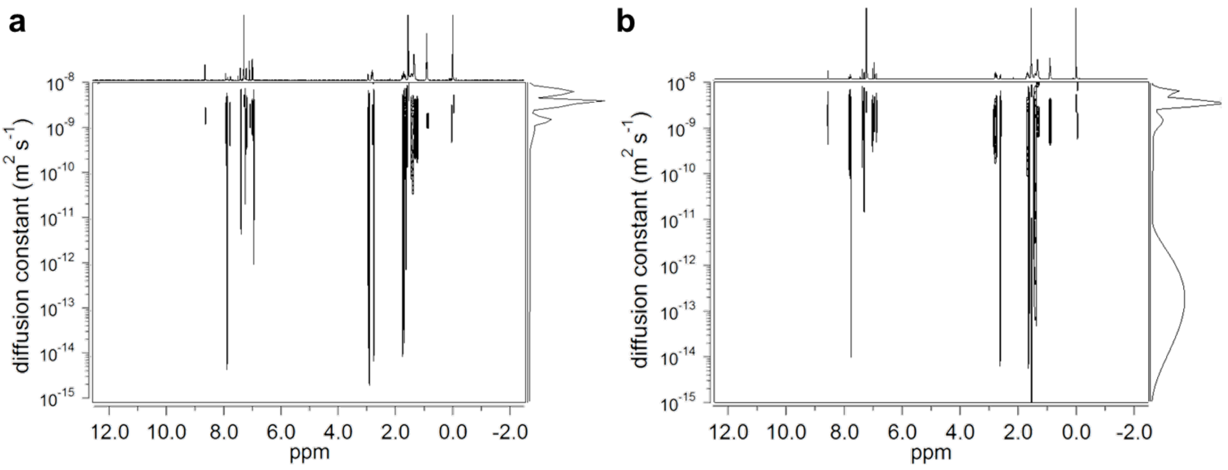

Figure S14. DOSY spectra of **1** (**a**) and **2** (**b**) in $CDCl_3$ at c = 1 mM.

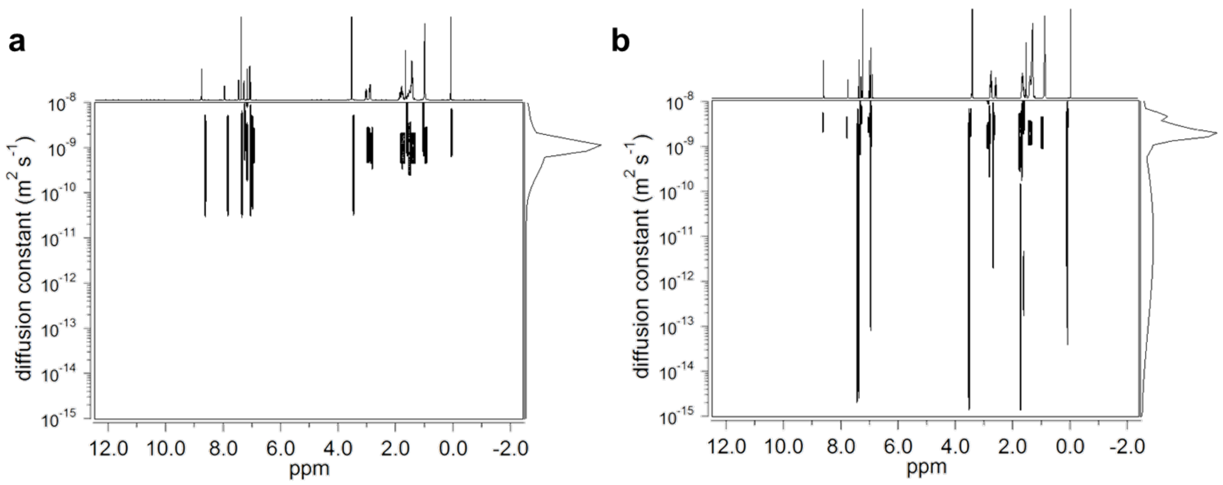

Figure S15. DOSY spectra of **1-Me** (**a**) and **2-Me** (**b**) in $CDCl_3$ at c = 10 mM.

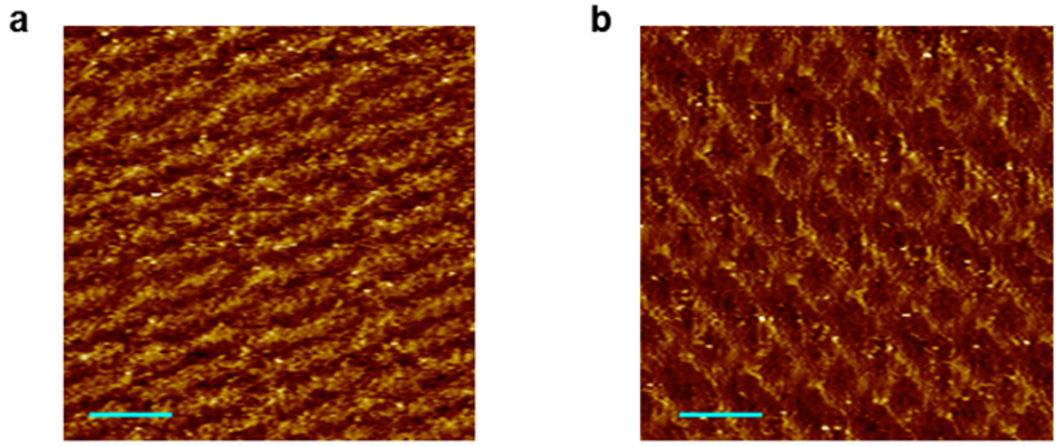

Figure S16. STM images of **1-Me** (**a**) and**2-Me** (**b**) at 1-phenyloctane–HOPG interface. The tunneling conditions: (**a**) I = 3.0 pA, V = −480 mV; (**b**) I = 1.6 pA, V = −600 mV. Scale bar, 3 nm.

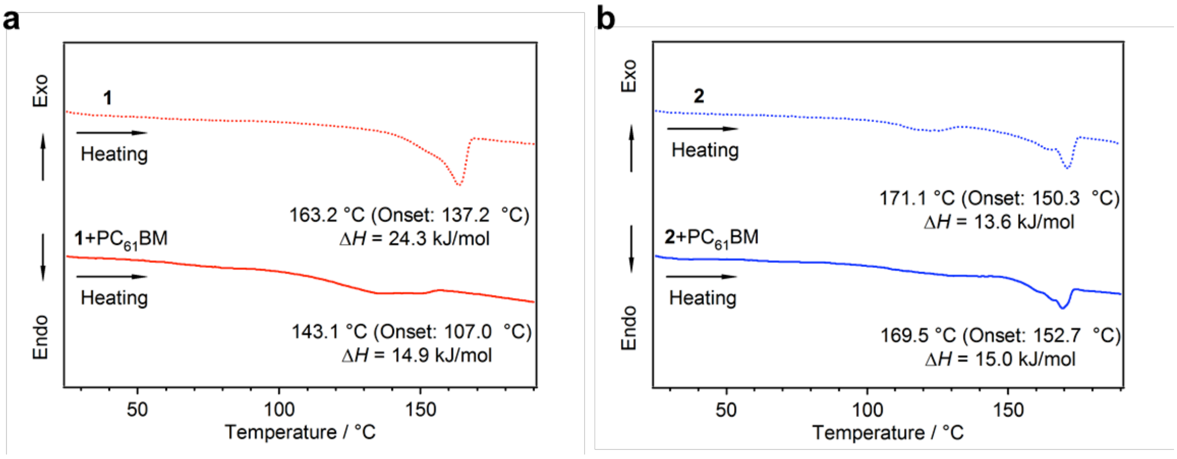

Figure S17. DSC profiles (first heating) of **1** (broken curve) and **1**:$PC_{61}BM$ (solid curve) (**a**) and **2** (broken curve) and **2**:$PC_{61}BM$ (solid curve) (**b**) measured at a scan rate of 5 °C/min. All samples were prepared by drop-casting $CHCl_3$ solutions onto a glass plate to form thin films. After drying at room temperature for 24 h to remove the residual solvent, the films were collected by scratching and transferred to an aluminum sample pan for DSC measurements.

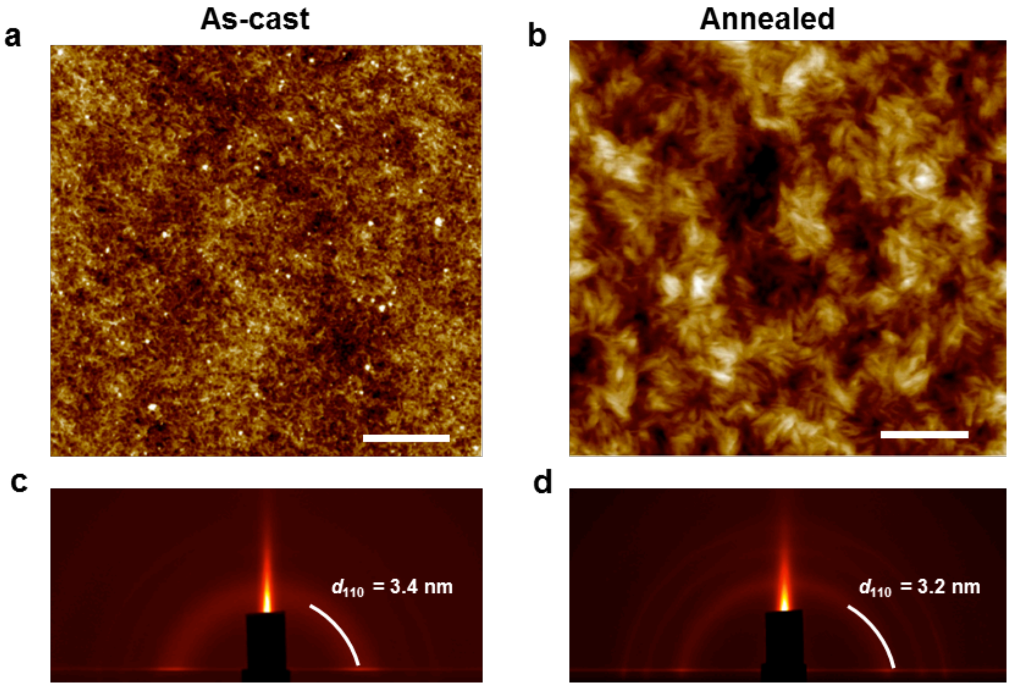

Figure S18. AFM (**a**,**b**) and GI-XRD (**c**,**d**) images of as-cast (**a**,**c**) and annealed (**b**,**d**) thin films of **1**. Scale bar, 200 nm. Thin film samples were prepared by spin-coating $CHCl_3$ solutions of **1** (c = 50 mM) onto silicon substrates. Annealing conditions: T = 80 °C, t = 10 min.

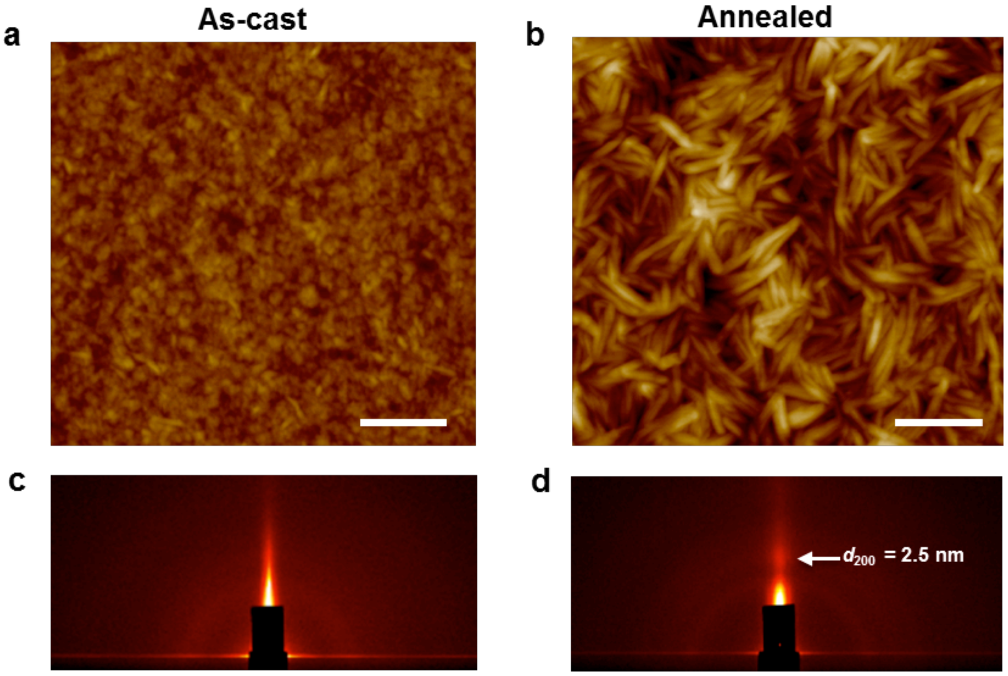

Figure S19. AFM (**a**,**b**) and GI-XRD (**c**,**d**) images of as-cast (**a**,**c**) and annealed (**b**,**d**) thin films of **1**:$PC_{61}BM$. Scale bar, 200 nm. Samples were prepared by spin-coating $CHCl_3$ solutions of the mixture (c_{total} = 20 mg mL^{-1}) onto silicon substrates. Annealing conditions: T = 80 °C, t = 10 min

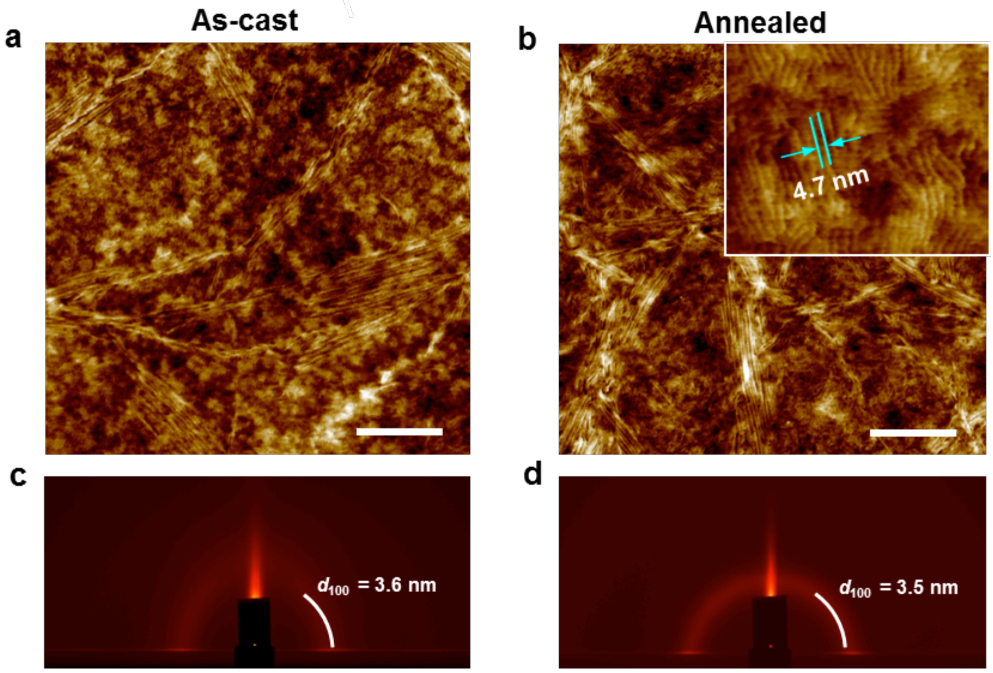

Figure S20. AFM (**a**,**b**) and GI-XRD (**c**,**d**) images of as-cast (**a**,**c**) and annealed (**b**,**d**) thin films of **2**. Scale bar, 200 nm. Thin film samples were prepared by spin-coating $CHCl_3$ solutions of **2** (c = 50 mM) onto silicon substrates. Annealing conditions: T = 80 °C, t = 10 min.

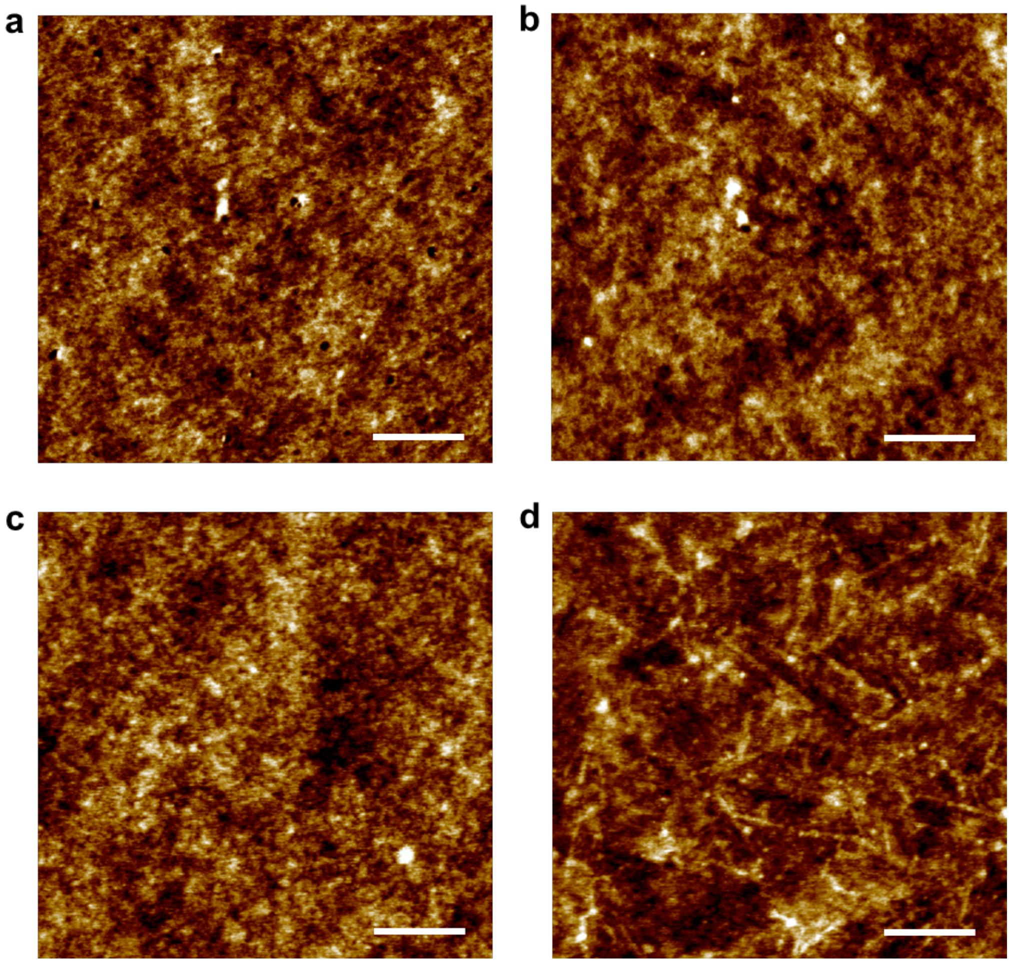

Figure S21. AFM images of 1:1 (w:w) blend films of **1-Me**:$PC_{61}BM$ (**a**,**c**) and **2-Me**:$PC_{61}BM$ (**b**,**d**) before (**a**,**b**) and after annealing at 80 °C (**c**,**d**). Scale bar, 200 nm.

Table S1. Comparison of the electronic properties of **1**, **2**, **1-Me**, and **2-Me**.

materials	E_g (eV)	E_{HOMO} (eV)	E_{LUMO} (eV)
**1**	1.84	–5.06	–3.22
**2**	1.78	–5.10	–3.32
**1-Me**	1.74	–5.11	–3.37
**2-Me**	1.78	–5.03	–3.25

Table S2. SCLC hole mobilities of **1** and **2**.

materials	annealing temp. (°C)	hole mobility (10^{-5} cm^2 V^{-1} s^{-1})
**1**	as-cast	6.5
	80	12.9
**2**	as-cast	17.1
	80	1.1

Table S3. Performance of BHJ solar cells of **1**:$PC_{61}BM$ and **2**:$PC_{61}BM$.

BHJ films	annealing temp. (°C)	V_{oc} (V)	J_{sc} (mA cm^{-2})	FF (%)	PCE (%)
**1**:$PC_{61}BM$	as-cast	0.88 ± 0.03	2.97 ± 0.06	28.9 ± 0.6	0.76 ± 0.04
	50	0.80 ± 0.09	2.53 ± 0.04	28.4 ± 1.1	0.58 ± 0.09
	80	0.77 ± 0.01	5.18 ± 0.00	37.8 ± 0.6	1.51 ± 0.04
	110	0.74 ± 0.03	2.67 ± 0.04	35.6 ± 0.3	0.70 ± 0.02
**2**:$PC_{61}BM$	as-cast	0.85 ± 0.01	2.20 ± 0.01	41.6 ± 0.6	0.78 ± 0.02
	50	0.85 ± 0.01	2.21 ± 0.02	40.2 ± 0.3	0.75 ± 0.02
	80	0.79 ± 0.01	1.30 ± 0.03	33.2 ± 0.1	0.34 ± 0.01
	110	0.69 ± 0.01	1.74 ± 0.02	38.5 ± 0.3	0.46 ± 0.02

Table S4. Performance of BHJ solar cells of **1-Me**:$PC_{61}BM$ and **2-Me**:$PC_{61}BM$.

BHJ films	annealing temp. (^{o}C)	V_{oc} (V)	J_{sc} (mA cm^{-2})	FF (%)	PCE (%)
**1-Me**:$PC_{61}BM$	as-cast	0.84 ± 0.06	3.75 ± 0.06	25.5 ± 0.2	0.8 ± 0.08
	50	0.63 ± 0.10	2.24 ± 0.03	25.5 ± 0.4	0.40 ± 0.09
	80	0.83 ± 0.07	4.00 ± 0.02	25.5 ± 0.3	0.85 ± 0.06
	110	0.85 ± 0.02	1.39 ± 0.01	24.6 ± 0.1	0.29 ± 0.01
**2-Me**:$PC_{61}BM$	as-cast	0.85 ± 0.01	231 ± 0.01	26.0 ± 0.2	0.51 ± 0.01
	50	0.85 ± 0.04	2.74 ± 0.04	31.3± 1.0	0.73 ± 0.06
	80	0.79 ± 0.01	1.84 ± 0.02	25.0 ± 0.2	0.37 ± 0.01
	110	0.76 ± 0.11	1.34 ± 0.06	24.4 ± 0.4	0.25 ± 0.05

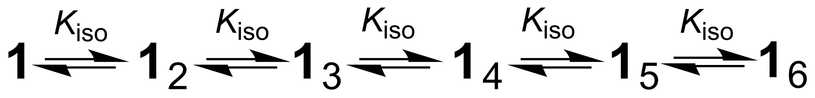

Scheme S1. Formation of rosette $\mathbf{1}_6$ by isodesmic binding of **1** with K_{iso}.

Supplementary References

S1. Yagai, S. *et al*. Supramolecular engineering of oligothiophene nanorods without insulators: hierarchical association of rosettes and photovoltaic properties. *Chem. Eur. J.* **20**, 16128−16137 (2014).
